# Hydrogen Peroxide Assisted Electrooxidation of Benzene to Phenol over Bifunctional Ni–(O–C_2_)_4_ Sites

**DOI:** 10.1002/advs.202204043

**Published:** 2022-10-30

**Authors:** Shengbo Zhang, Meng Jin, Hui Xu, Wenyi Li, Yixing Ye, Tongfei Shi, Hongjian Zhou, Chun Chen, Guozhong Wang, Yunxia Zhang, Yue Lin, Lirong Zheng, Haimin Zhang, Huijun Zhao

**Affiliations:** ^1^ Key Laboratory of Materials Physics Centre for Environmental and Energy Nanomaterials Anhui Key Laboratory of Nanomaterials and Nanotechnology CAS Center for Excellence in Nanoscience Institute of Solid State Physics HFIPS Chinese Academy of Sciences Hefei 230031 China; ^2^ University of Science and Technology of China Hefei 230026 China; ^3^ Hefei National Research Center for Physical Sciences at the Microscale University of Science and Technology of China Hefei 230026 China; ^4^ Beijing Synchrotron Radiation Facility Institute of High Energy Physics Chinese Academy of Sciences 19B Yuquan Road Beijing 100049 China; ^5^ Centre for Catalysis and Clean Energy Griffith University Gold Coast Campus QLD 4222 Australia

**Keywords:** 2e^−^ ORR, benzene oxidation, Ni–O coordination, phenol, single‐atom catalysts

## Abstract

Direct electrocatalytic oxidation of benzene has been regarded as a promising approach for achieving high‐value phenol product, but remaining a huge challenge. Here an oxygen‐coordinated nickel single‐atom catalyst (Ni–O–C) is reported with bifunctional electrocatalytic activities toward the two‐electron oxygen reduction reaction (2e^−^ ORR) to H_2_O_2_ and H_2_O_2_‐assisted benzene oxidation to phenol. The Ni–(O–C_2_)_4_ sites in Ni–O–C ar proven to be the catalytic active centers for bifunctional 2e^−^ ORR and H_2_O_2_‐assisted benzene oxidation processes. As a result, Ni–O–C can afford a benzene conversion as high as 96.4 ± 3.6% with a phenol selectivity of 100% and a Faradaic efficiency (FE) of 80.2 ± 3.2% with the help of H_2_O_2_ in 0.1 m KOH electrolyte at 1.5 V (vs RHE). A proof of concept experiment with Ni–O–C concurrently as cathode and anode in a single electrochemical cell demonstrates a benzene conversion of 33.4 ± 2.2% with a phenol selectivity of 100% and a FE of 44.8 ± 3.0% at 10 mA cm^−2^.

## Graphical abstract

For the first time an oxygen‐coordinated nickel single‐atom catalyst is demonstrated with bifunctional electrocatalytic activities toward the two‐electron oxygen reduction reaction to H_2_O_2_ and H_2_O_2_‐assisted benzene oxidation to phenol. Taking the advantage of operando SR‐FTIR and operando Raman measurements, the early C–H activation mechanism i verified.

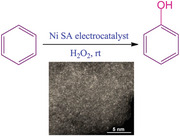

## 1 Introduction

Phenol is a very important industrial chemical in the manufacture of phenolic resin, pharmaceuticals, agrochemicals, and dyes etc.^[^ ^1^, ^2^ ^]^ In 2021, the global phenol market is approximate to 15 million tonnes per year. Up to now, industrial scale phenol production is still heavily dependent on the well‐known cumene method with complex reaction process under high temperature, high pressure, and strongly acidic conditions.^[^ ^3^, ^4^ ^]^ In addition, this three‐step cumene process possesses a low overall yield of phenol (<5%) and concurrently generates a large quantity of acetone as a by‐product (production of 1 tonne phenol accompanying with 0.65 tonne acetone generation).^[^ ^5^, ^6^ ^]^ Therefore, researchers have been pursuing the development of new phenol production techniques, such as one‐step hydroxylation of benzene with the help of various oxidants (e.g., N_2_O, H_2_O_2_, O_2_ etc.) and catalysts under the elevated temperature and pressure conditions.^[^ ^7^, ^8^, ^9^ ^]^ Although considerable achievements have been obtained from the fundamental and applied researches, one‐step hydroxylation of benzene to phenol by such directly catalytic oxidation approach is still confronting big obstacles to large‐scale industrial applications, due to its high energy consumption, high cost, and low efficiency.^[^ ^7^, ^8^, ^9^ ^]^ Therefore, it is still highly desirable for the development of environmentally friendly and high‐efficiency directly catalytic oxidation of benzene to phenol technique at ambient conditions.

Renewable‐energy‐powered electrocatalysis has been reported as an attractive approach to the production of phenol by direct electrocatalytic oxidation of benzene with the help of molecule O_2_.^[^ ^10^ ^]^ In 2012, Hibino and co‐workers reported the utilization of different metal oxides, such as V_2_O_5_, Mn_2_O_3_, CoO, CuO, Fe_2_O_3_, MoO_3_, MgO, WO_3_, ZrO_2_, and Cr_2_O_3_, as the anode materials for directly electrocatalytic oxidation of benzene to phenol through an vapour‐phase electrocatalytic approach at 50 °C.^[^ ^11^ ^]^ The results demonstrated that the V_2_O_5_ anode exhibits the best electrocatalytic activity among all anodes investigated with the current efficiency of 41.7% and selectivity of 100% for phenol production under the assistance of molecule O_2_.^[^ ^11^ ^]^ The reported works have suggested that the active oxygen species (AOSs), such as *O, ·HO, ·HOO, O_2_^2−^, O^−^, are responsible for benzene oxidation to phenol during catalysis.^[^ ^12^ ^]^ Therefore, to further improve the electrosynthesis efficiency of phenol, it is rational to design electrocatalysts possessing high‐density active sites with the capabilities of AOSs generation and adsorption and activation of benzene. It is well known that AOSs (e.g., *O, ·HO, ·HOO, O_2_^2−^, O^−^) can be effectively generated by the electrooxidation of H_2_O_2_,^[^ ^11^, ^13^ ^]^ moreover H_2_O_2_ can be efficiently synthesized by a two‐electron oxygen reduction reaction (2e^−^ ORR) via electrocatalysis.^[^ ^14^, ^15^ ^]^ This inspired us to develop a multifunctional electrocatalyst integrated into an electrochemical reaction system, enabling electrocatalytic 2e^−^ ORR to H_2_O_2_ as the cathode catalyst, and concurrent electrooxidation of H_2_O_2_ to AOSs and hydroxylation of benzene to phenol as the anode catalyst. However, similar concept has not been reported in literatures. Recently, nickel‐based single‐atom (SA) catalysts (Ni–SA) with Ni–N_2_O_2_ and Ni–N_4_ moieties have exhibited superior H_2_O_2_ yield and selectivity during electrocatalytic 2e^−^ ORR.^[^ ^16^, ^17^ ^]^ Therefore, it is vital for design and development of Ni–SA catalyst with new coordination configuration as catalytic active sites for high‐efficiency electrocatalytic hydroxylation of benzene to phenol at ambient conditions.

In this work, we report an adsorption‐regulated approach to synthesize oxygen‐coordinated Ni–SA sites on carbon (denoted as Ni–O–C) as efficient electrocatalyst for electrochemical oxidation of benzene to phenol with the assistance of H_2_O_2_ at ambient conditions. The results demonstrated that the obtained Ni–O–C exhibits superior bifunctional electrocatalytic activities toward the 2e^−^ ORR to H_2_O_2_ and H_2_O_2_‐assisted benzene oxidation to phenol. Concretely, the Ni–O–C can afford a benzene conversion as high as 96.4 ± 3.6% with a phenol selectivity of 100% and a Faradaic efficiency (FE) of 80.2 ± 3.2% in the presence of H_2_O_2_ in 0.1 m KOH electrolyte at 1.5 V (vs RHE). Moreover, the Ni–O–C also indicates superior 2e^−^ ORR to H_2_O_2_ activity with a high H_2_O_2_ yield rate of 1.08 ± 0.03 mol g_cat._^−1^ h^−1^ and a FE of 92.5 ± 3.3% at 0.4 V (vs RHE) in 0.1 m KOH, surpassing most of state‐of‐the‐art 2e^−^ ORR electrocatalysts reported in literatures (Table S1, Supporting Information). The spectroscopic studies and theoretical calculations results unveiled that the Ni–(O–C_2_)_4_ sites in Ni–O–C are the catalytic active centers for 2e^−^ ORR to H_2_O_2_ and H_2_O_2_‐assisted benzene oxidation to phenol. In addition, a proof of concept experiment with Ni–O–C concurrently as cathode and anode in a single electrochemical cell exhibited a benzene conversion of 33.4 ± 2.2% with a phenol selectivity of 100% and a FE of 44.8 ± 3.0% at a current density of 10 mA cm^−2^

## 2 Results and Discussion

### 2.1 Synthesis and Characteristics of Ni–O–C

**Figure 1:**
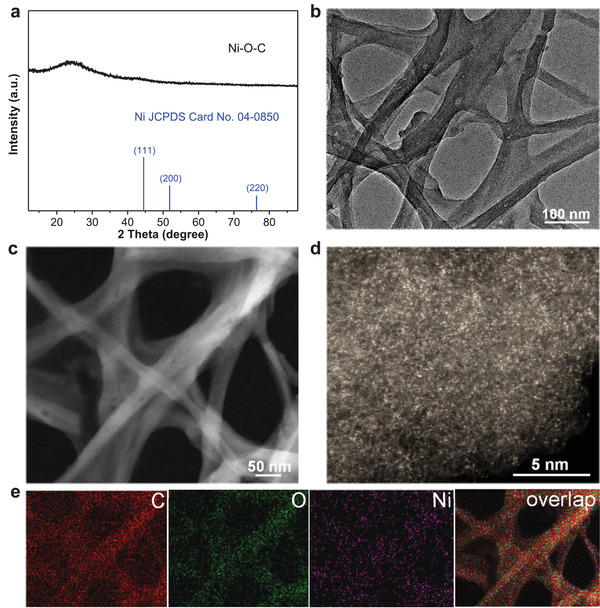
Synthesis of Ni SAs. a) XRD patterns and b) TEM image of Ni–O–C. c) Low‐ and d) high‐magnification aberration‐corrected HAADF‐STEM images of Ni–O–C. e) The corresponding elemental mapping images of Ni–O–C.

The oxygen‐coordinated nickel single‐atom (SA) catalyst (Ni–O–C) was synthesized using a similar synthetic method reported in our previous works.^[^ ^18^, ^19^ ^]^ In the synthetic procedure, the pretreated bacterial cellulose (BC) with rich oxygen groups and nanofiber network structures (Figure S1, Supporting Information) was used as the adsorption regulator to impregnate Ni^2+^ with an initial concentration of 240 mmol L^−1^. After adequate adsorption of 6 h (Figure S2, Supporting Information), the obtained sample was denoted as Ni^2+^–BC with a Ni content of ≈12.3 wt%, followed by a carbothermal reduction treatment to synthesize metallic Ni nanoparticles supported on the carbonized BC (Ni–CBC). The as‐synthesized Ni–CBC exhibited metallic Ni structure (JCPDS Card No. 04–0850) (Figure S3a, Supporting Information), and the electron microscope images showed fiber‐like carbon structure supported metallic Ni (111) nanoparticles (Figure S3b,c, Supporting Information) with a Ni content of ≈51.0 wt%. After adequate acid‐etching treatment, the Ni content in Ni–CBC was dramatically reduced to be ≈1.04 wt%, meaning effective removal of metallic Ni nanoparticles to possibly form atomically dispersed Ni–O–C. After the acid‐etching process, the XRD patterns of Ni–O–C only displayed the characteristic diffraction peaks assignable to the (002) and (101) planes of graphitic carbon, and no metallic Ni nanoparticles were checked on carbon (**Figure** **1**a,b). The aberration‐corrected high‐angle annular dark‐field scanning transmission electron microscopy (HAADF‐STEM) image (Figure 1c) further confirmed this, and disclosed the existence of densely populated and atomically dispersed bright dots on the acid‐etching sample, implying that the remaining Ni in the sample is in the form of single atoms supported on carbon (Figure 1d). The presence of Ni–SA sites can be further evidenced by the aberration‐corrected HAADF‐STEM images took from different locations of Ni–O–C (Figure S4, Supporting Information). The corresponding elemental mapping analysis revealed that C, O, and Ni elements are homogeneously distributed over the entire Ni–O–C (Figure 1e). The Raman spectrum (Figure S5, Supporting Information) showed distinctive peaks assignable to the D and G bands of graphite carbon of Ni–O–C.^[^ ^18^, ^19^ ^]^ The N_2_ adsorption–desorption isotherm measurement indicated that the Ni–O–C possesses a Brunauer–Emmett–Teller (BET) specific surface area of 647.3 m^2^ g^−1^ with micro‐ and mesoporous structures (Figure S6, Supporting Information), beneficial for the exposure of active sites and mass transport during electrocatalysis.^[^ ^18^, ^19^ ^]^

### 2.2 Confirmation of Ni–(O–C_2_)_4_ SA Sites

**Figure 2:**
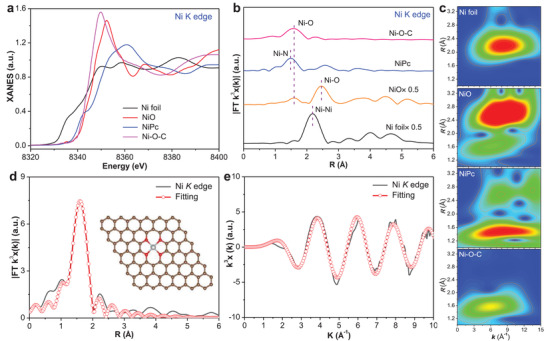
Ni–(O–C_2_)_4_ site configuration. Ni *K* edge a) XANES spectra and b) *k* ^3^‐weighted FT‐EXAFS spectra of Ni–O–C and references. c) Ni *K* edge WT‐EXAFS of Ni–O–C and references. Ni *K* edge EXAFS fitting curves of Ni–O–C at d) *R* space and e) *k* space. Inset in (d) is the proposed Ni–(O–C_2_)_4_ coordination configuration in Ni–O–C (Brown: C, red: O, White: Ni).

It is well known that the coordination environment of SA catalysts is critically important for their high catalytic activities.^[^ ^20^, ^21^ ^]^ The X‐ray photoelectron spectroscopy (XPS) survey spectrum of Ni–O–C confirmed the presence of Ni, O, and C elements (Figure S7a, Supporting Information). The C 1s (Figure S7b, Supporting Information) and O 1s XPS spectra (Figure S7c, Supporting Information) verified the existence of rich O groups and the formation of Ni–O bond in Ni–O–C.^[^ ^18^, ^19^ ^]^ The Ni 2p spectrum displayed the binding energies of Ni 2p_3/2_ and Ni 2p_1/2_ peaks at 858.4 and 878.8 eV with two satellite peaks at 861.8 and 882.2 eV, respectively, which are the characteristic of Ni^2+^ (Figure S7d, Supporting Information).^[^ ^22^ ^]^ Although the XPS characterizations have confirmed the presence of Ni–O bond in Ni–O–C, the exact coordination structure of oxygen‐coordinated Ni–SA sites on carbon is still unclear. Therefore, the synchrotron‐based X‐ray absorption near‐edge structure (XANES) and the extended X‐ray absorption fine structure (EXAFS) spectra of Ni K‐edge in Ni–O–C were measured to further disclose the electronic structure and chemical configuration of Ni–O–C. The Ni adsorption edge positions of Ni–O–C are essentially consistent with those of NiO standard, indicating that the average oxidation state of Ni in Ni–O–C is close to Ni^2+^ (**Figure** **2**a).^[^ ^16^, ^17^ ^]^ The Fourier transform (FT) EXAFS spectra demonstrated that Ni–O–C exhibits only one obvious FT peak located at ≈1.6 Å, mainly attributed to the scattering of Ni‐O coordination (Figure 2b). For the Ni foil and nickel porphyrin (NiPc) references, Ni–Ni and Ni–N coordination derived scattering peaks can be clearly observed at ≈2.2 and ≈1.5 Å (Figure 2b), respectively, apparently different to that derived from Ni–O coordination in Ni–O–C. This can be further verified by the *q* space magnitudes for *k* ^3^‐weighted FT‐EXAFS paths (Figure S8, Supporting Information). The Ni K‐edge wavelet transform (WT)‐EXAFS spectra (Figure 2c) were also obtained to further illustrate the bonding states of the adjacent metal atoms in Ni–O–C. As shown in Figure 2c, the WT contour plots of Ni–O–C displayed only one intensity maximum at ≈6.0 Å^−1^, corresponding to the Ni–O coordination. Additionally, the WT signals related to Ni–Ni contribution at ≈8.2 Å^−1^ and Ni–N contribution at ≈8.4 Å^−1^ were not detected in Ni–O–C (Figure 2c). The above characterizations results confirmed the existence of Ni–O coordination in Ni–O–C. Subsequently, the quantitative EXAFS fitting was performed to extract the structural parameters, and the fitting results were shown in Figure 2d,e and Table S2 (Supporting Information). The first shell of the central atom Ni displayed a coordination number of four. Based on the EXAFS fitting results, it can be speculated that the atomically dispersed Ni–SA sites on carbon in Ni–O–C are constructed through one Ni atom coordinated with four oxygen atoms, namely, the Ni–(O–C_2_)_4_ is the most likely SA site in Ni–O–C (inset of Figure 2d).

### 2.3 H_2_O_2_‐Assisted Benzene Oxidation to Phenol on Anode

**Figure 3:**
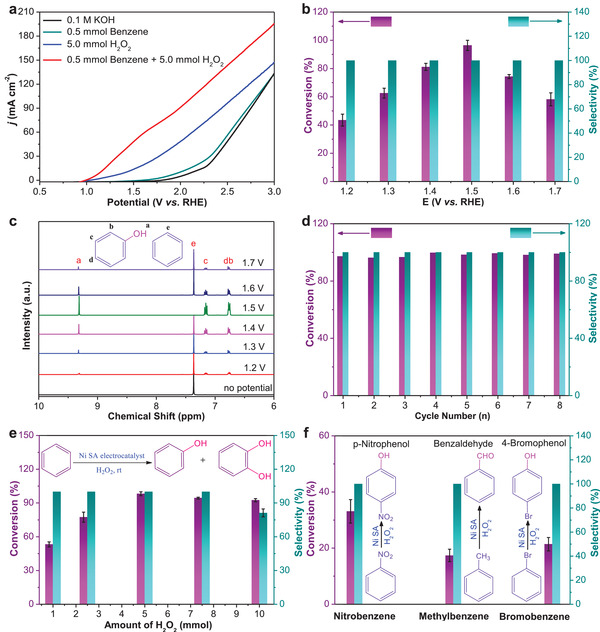
Electrochemical oxidation of benzene. a) LSV curves of Ni–O–C anode in 0.1 m KOH electrolyte with and without 0.5 mmol benzene, 5.0 mmol H_2_O_2_, and 0.5 mmol benzene + 5.0 mmol H_2_O_2_. b) Dependence of the conversion of benzene and the selectivity of phenol on applied potentials. c) The ^1^H NMR spectra of the products corresponding to the applied potentials. d) Cycling stability test of electrochemical oxidation of benzene at 1.5 V (vs RHE) for 8 cycles. e) Effect of H_2_O_2_ amount on benzene oxidation catalyzed by Ni–O–C at 1.5 V (vs RHE). f) Electrocatalytic oxidation of nitrobenzene, methylbenzene, and bromobenzene using Ni–O–C at 1.5 V (vs RHE).

The reported works have demonstrated that the electrocatalytic oxidation of benzene to phenol is very feasible with the help of active oxygen species (AOSs) generated at cathode or anode.^[^ ^10^, ^11^, ^12^, ^23^, ^24^, ^25^ ^]^ Although the phenol selectivity is approached to 100% in their studies, the current efficiency for phenol production is low (the highest current efficiency of 41.7% on V_2_O_5_ anode).^[^ ^11^ ^]^ In this work, we expected that the AOSs can be generated by H_2_O_2_ oxidation on Ni–O–C anode, which enable direct hydroxylation of benzene to phenol on the same anode. For this, we evaluated the electrocatalytic activity toward benzene oxidation reaction using an H‐type two‐compartment cell consisting of a three‐electrode electrochemical system with a Ni–O–C based working electrode, an Ag/AgCl (saturated KCl solution) reference electrode and a Pt mesh counter electrode. The electrolyte was 0.1 m KOH aqueous solution, and Ar gas was persistently bubbled into the electrolyte to eliminate O_2_ interference during electrocatalytic oxidation of benzene. First, the linear sweep voltammetry (LSV) curves of Ni–O–C were measured in Ar‐saturated 0.1 m KOH without or with 0.5 mmol benzene, 5.0 mmol H_2_O_2_, and 0.5 mmol benzene + 5.0 mmol H_2_O_2_. As shown in **Figure** **3**a, the anodic current densities of Ni–O–C in the investigated potential range are attributed to the oxygen evolution reaction (OER) in Ar‐saturated 0.1 m KOH without the introduction of benzene and/or H_2_O_2_. Comparatively, when only 0.5 mmol benzene was added to Ar‐saturated 0.1 m KOH, a slight increase in anodic current densities was observed. Interestingly, it was found that when only 5.0 mmol H_2_O_2_ was introduced to the electrolyte, the anodic current densities in the LSV curve of Ni–O–C were dramatically enhanced with an obvious negative shift of the onset potential, ascribed to high‐efficiency electrooxidation of H_2_O_2_ on Ni–O–C to generate AOSs.^[^ ^11^ ^]^ The formed AOSs are highly beneficial for subsequently electrocatalytic oxidation of benzene to phenol. This can be further validated by the LSV measurement with the addition of 0.5 mmol benzene + 5.0 mmol H_2_O_2_ into the electrolyte, exhibiting significantly improved anodic current densities compared to those obtained in only H_2_O_2_‐introduced electrolyte. The above LSV measurements results indicated superior electrocatalytic oxidation activity of Ni–O–C toward benzene to phenol in the presence of H_2_O_2_ and the onset potential for benzene oxidation was ≈ 1.0 V (vs RHE). Figure S9 (Supporting Information) shows the chronoamperometric curves of Ni–O–C at different potentials with 2 h of reaction for each measurement in 0.5 mmol benzene + 5.0 mmol H_2_O_2_ introduced Ar‐saturated 0.1 m KOH electrolyte. After 2 h of electrocatalysis, the electrolyte was first acidified and then concentrated by the rotary evaporation, the obtained products were finally extracted with ethyl acetate for further qualitative and quantitative analysis (Figure S10, Supporting Information). The gas chromatography (GC) measurements results (Figure S11, Supporting Information) demonstrated that except for the signal peaks of ethyl acetate, only benzene signal peak was detectable without the anodic potential, while phenol product signal peaks were clearly observed owing to the benzene oxidation at different anodic potentials. Moreover, with increasing the potential from 1.2 to 1.7 V (vs RHE), the signal peak intensity of benzene initially decreased and almost no benzene signal was detected at the potential of 1.5 V (vs RHE). Correspondingly, the signal peak intensity of phenol product initially increased with the applied potential, which reached the maximum (0.48 ± 0.02 mmol) at 1.5 V (vs RHE), meaning that benzene was almost completely converted to phenol at 1.5 V (vs RHE). When the potential was employed to be more positive, the electrocatalytic conversion of benzene was obviously decreased, primarily attributed to the competitive oxygen evolution reaction (OER) concurrently happened on Ni–O–C.^[^ ^26^ ^]^ Figure 3b shows the dependence of the conversion of benzene and the selectivity of phenol on the potentials in 0.5 mmol benzene + 5.0 mmol H_2_O_2_ introduced Ar‐saturated 0.1 m KOH. As can be seen that under the given experimental conditions, phenol is the only product through electrocatalytic oxidation of benzene with almost 100% selectivity at all investigated potentials. However, the conversion of benzene is highly dependent on the applied potential, and a superb benzene conversion of 96.4 ± 3.6% with 100% phenol selectivity and a faradaic efficiency (FE) of 80.2 ± 3.2% was attained at 1.5 V (vs RHE). To the best of our knowledge, the achieved benzene conversion and phenol selectivity of Ni–O–C in this work are the highest among all electrocatalysts reported to date (Table S3, Supporting Information).^[^ ^10^, ^11^, ^12^, ^23^, ^24^, ^25^ ^]^ The effect of electrolyte pH was also examined (Figure S12, Supporting Information) using 0.05 m H_2_SO_4_ electrolyte (pH = 1.5), 0.1 m Na_2_SO_4_ electrolyte (pH = 6.3), and 0.1 m KOH electrolyte (pH = 12.8) at 1.5 V (vs RHE). The highest benzene conversion were obtained from 0.1 m KOH electrolyte (pH = 12.8), suggesting a superior electrocatalytic activity of Ni–O–C in alkaline electrolyte. For the sake of data reliability, the products from electrocatalytic oxidation of benzene were further confirmed by the ^1^H nuclear magnetic resonance (^1^H NMR) measurements. As shown in Figure 3c, no potential was applied, only the signal peak at ≈7.4 ppm of benzene ring in benzene was detected. With applying the potential up to the optimum 1.5 V (vs RHE), the signal peak of benzene ring in benzene was completely disappeared, while the signal peaks of phenolic hydroxyl at ≈9.3 ppm and benzene ring in phenol at ≈7.2 and ≈6.8 ppm reached the largest intensities among all investigated potentials (Figure S13, Supporting Information), indicating almost complete conversion of benzene to phenol by Ni–O–C electrocatalysis with almost 100% selectivity. The above ^1^H NMR results are consistent with the GC measurements results. The controllable experiments were conducted in 0.5 mmol benzene introduced Ar‐saturated 0.1 m KOH electrolyte without H_2_O_2_ (Figure S14a, Supporting Information). As shown in Figure S14b (Supporting Information), only the signal peak at ≈7.4 ppm of benzene ring in benzene can be detected and no signal peaks of phenol are detectable after electrocatalysis from 1.2 to 1.7 V (vs RHE) for 2 h. These experimental results exclude the likelihood of benzene oxidation mediated directly by Ni–(O–C_2_)_4_ without H_2_O_2_, indicating the important role of H_2_O_2_ for electrocatalytic oxidation of benzene to phenol. The chronoamperometric stability of Ni–O–C was examined in 0.1 m KOH electrolyte with 5.0 mmol benzene + 50.0 mmol H_2_O_2_ at 1.5 V (vs RHE) over a 10 h period for three replicated experiments (Figure S15, Supporting Information). The obtained chronoamperometric curves from the three replicated experiments display ignorable changes in the current density over the entire test period, corresponding to an average benzene conversion as high as 97.2 ± 1.4% with a phenol selectivity of 100%, signifying superior benzene oxidation stability. The cycling stability of Ni–O–C for benzene oxidation was further evaluated at 1.5 V (vs RHE) with a testing period of 2 h for 8 consecutive cycles. Slight change in the chronoamperometric profiles was observed (Figure S16a, Supporting Information), which resulted in very stable benzene conversion with 100% phenol selectivity during 8 consecutive cycling measurements (Figure 3d; Figure S16b, Supporting Information), demonstrating superior cycling stability of Ni–O–C for electrocatalytic oxidation of benzene. The superb performance of Ni–O–C can be attributed to its structural stability as evidenced by the well retained atomically dispersed Ni species without aggregated metallic Ni nanoparticles after consecutive cycling tests (Figures S17–S21, Supporting Information). In this work, we have evaluated the energy efficiency of overall electrochemical cell in a three‐electrode system to investigate the effect of H_2_O_2_ on the electrochemical oxidation of benzene to phenol (Table S4, Supporting Information). As shown, with increasing the potential from 1.2 to 1.7 V (vs RHE), the practical applied potential (*∆E*) on the electrochemical cell is obviously increased, resulting in obviously decreased energy efficiency. This is mainly because at low potential (e.g., 1.2 V, vs RHE), the electrocatalytic oxidation reaction of benzene to phenol, including other side‐reactions, is thermodynamically inferior, while with increasing the potential, the decreased energy efficiency is mainly due to the side‐reaction of electrocatalytic H_2_O_2_ decomposition to O_2_ on the anode. At higher applied potentials, the electrochemical decomposition of H_2_O_2_ to O_2_ in the electrochemical system is very outstanding, resulting in decreased electrocatalytic oxidation efficiency of benzene to phenol.

In this work, hydrogen peroxide (H_2_O_2_) plays a vital role in the generation of active oxygen species (AOSs) for electrocatalytic oxidation of benzene to phenol on Ni–O–C. A suitable amount of H_2_O_2_ in electrolyte is critically important for high‐efficiency phenol production, because excess H_2_O_2_ in reaction system may cause excessive oxidation of phenol, thus resulting in the decreased selectivity.^[^ ^1^, ^2^, ^3^, ^27^, ^28^ ^]^ In this study, to evaluate the effect of H_2_O_2_ amount on the electrocatalytic oxidation of benzene to phenol, 0.5 mmol benzene was fixed in Ar‐saturated 0.1 m KOH electrolyte for electrochemical measurements. The LSV curves (Figure S22a, Supporting Information) of Ni–O–C showed that the anodic current densities were increased initially with the H_2_O_2_ amount at 1.5 V (vs RHE), and reached the maximum value in the investigated potential range when the H_2_O_2_ amount was 5.0 mmol, owing to high‐efficiency electrocatalytic oxidation of benzene to phenol with the help of AOSs generated from H_2_O_2_ on Ni–O–C with the optimal ratio of benzene to H_2_O_2_ under the given reaction system. Further increasing the amount of H_2_O_2_ to 7.5 and 10 mmol, the anodic current densities in their LSV curves were found to be obviously decreased, possibly ascribed to easier self‐decomposition of H_2_O_2_ at higher concentration and excessive oxidation of phenol to generate other by‐products.^[^ ^1^, ^2^, ^3^, ^27^, ^28^ ^]^ The chronoamperometric profiles also exhibited similar results, as shown in Figure S22b (Supporting Information). After 2 h of electrocatalysis at 1.5 V (vs RHE), the electrocatalytic conversion of benzene reached the maximum value of 98.2 ± 1.8% with 5.0 mmol H_2_O_2_, and further increasing the H_2_O_2_ amount to 7.5 and 10 mmol, the benzene conversion was slightly decreased to be 94.4 ± 0.8% and 92.5 ± 1.4%, respectively (Figure 3e). Interestingly, it was found that the selectivity of phenol resulted from the electrocatalytic oxidation of benzene was still approximate to 100% with the added H_2_O_2_ amount from 1.0 to 7.5 mmol, but the phenol selectivity was decreased to be 81.2 ± 3.5% with 10 mmol H_2_O_2_ (Figure 3e). This was attributed to the generation of catechol side‐product because of the excessive oxidation of phenol product on Ni–O–C surface with higher concentration of AOSs from H_2_O_2_ oxidation, evidenced by the GC and ^1^H NMR measurements (Figure S23, Supporting Information). To validate the general applicability of such H_2_O_2_‐assisted electrocatalytic oxidation approach, a series of aromatic compounds, such as nitrobenzene, methylbenzene, and bromobenzene, were selected as the reactants instead of benzene precursor for electrocatalytic oxidation evaluation. The electrochemical measurements of Ni–O–C were performed in Ar‐saturated 0.1 m KOH electrolyte containing 0.5 mmol aromatic compound + 5.0 mmol H_2_O_2_ at 1.5 V (vs RHE) with 2 h of reaction. The corresponding chronoamperometric profiles are shown in Figure S24 (Supporting Information). The GC and ^1^H NMR measurements results indicated that after 2 h of reaction at 1.5 V (vs RHE), the electrocatalytic oxidation of methylbenzene, nitrobenzene, and bromobenzene generated benzaldehyde, *p*‐nitrophenol and 4‐bromophenol with almost 100% selectivity, respectively (Figures S25–S27, Supporting Information). The conversion of methylbenzene, nitrobenzene, and bromobenzene reached 17.4 ± 2.2%, 33.1 ± 4.2%, and 21.4 ± 2.4% under the given experimental conditions, respectively (Figure 3f). Comparatively, lower electrocatalytic conversion of nitrobenzene and bromobenzene were attributed to the substitution of H on benzene ring by –NO_2_ and –Br. Due to superior electron absorption ability of these groups, the electron cloud density on benzene ring would be reduced, making the benzene ring more difficult to hydroxylation. For methylbenzene, the electron on benzene ring and C–H bond of methyl produced hyperconjugation, which made the electron between C and H deviated from H, resulting in methyl more active, and easy to be oxidized to aldehyde or even carboxyl.^[^ ^29^, ^30^ ^]^ It can be imagined that the electrocatalytic oxidation efficiencies of different aromatic compounds aforementioned can be further improved through further optimizing the experimental parameters, such as the applied anodic potential, H_2_O_2_ concentration, and reaction time etc. The above results validated the feasibility of electrocatalytic oxidation of aromatic compounds with different functional groups in the presence of H_2_O_2_ for synthesizing their corresponding high‐value chemicals.

### 2.4 2e^−^ ORR to H_2_O_2_ on Cathode

The aforementioned experiments have demonstrated that H_2_O_2_ plays very important role in electrocatalytic oxidation of benzene to phenol at ambient conditions. H_2_O_2_ as an important industrial chemical is generally produced at industrial‐scale through the indirect and complex anthraquinone process.^[^ ^31^ ^]^ Undoubtedly, to employ H_2_O_2_ as a general chemical reagent for wide synthetic applications, development of renewable‐energy‐driven techniques for H_2_O_2_ synthesis is highly desirable. It has been documented that the electrocatalysis through two‐electron oxygen reduction reaction (2e^−^ ORR) has exhibited great potential for H_2_O_2_ synthesis.^[^ ^14^, ^15^ ^]^ Moreover, nickel‐based single‐atom (SA) catalysts (Ni–SA) with Ni–N_2_O_2_ and Ni–N_4_ moieties have recently demonstrated superior electrocatalytic 2e^−^ ORR activity toward H_2_O_2_ production.^[^ ^16^, ^17^ ^]^ In this work, the electrocatalytic ORR performance of Ni–O–C was evaluated in 0.1 m KOH electrolyte using the typical rotating ring‐disk electrode (RRDE) technique. Prior to all electrochemical tests, the collection efficiency of ring electrode was calibrated using K_3_[Fe(CN)_6_] solution and the value was determined to be 0.357, which is very approximate to the theoretical value of 0.370 (Figure S28, Supporting Information).^[^ ^14^, ^15^ ^]^ Figure S29a (Supporting Information) shows the LSV curves collected at 1600 rpm in O_2_‐saturated 0.1 m KOH, together with the H_2_O_2_ oxidation current collected by Pt ring electrode at a constant potential of 1.2 V (vs RHE). The calculated H_2_O_2_ selectivity and electron transfer number (*n*) are plotted in Figure S29b (Supporting Information). The Ni–O–C showed a high H_2_O_2_ selectivity of 85–90% and the *n* value was calculated to be less than 2.3 in a wide potential range of 0.2–0.6 V (vs RHE), revealing a selective 2e^−^ ORR pathway. Furthermore, we also carried out the RRDE tests at different rotation speeds (Figure S29c, Supporting Information) in 0.1 m KOH, and used the Koutecky–Levich (*K–L*) equations to calculate the electron transfer number (*n*) during electrocatalytic ORR process (Figure S29d, Supporting Information). The results indicated that the *n* value was calculated to be 2.33 at 0.3 V, 2.33 at 0.4 V, and 2.36 at 0.5 V (vs RHE), respectively, further suggesting a 2e^−^ ORR process beneficial for H_2_O_2_ formation. The long‐term stability of Ni–O–C was further assessed by the chronoampermetric test at a constant disk potential of 0.4 V (vs RHE) for 12 h (Figure S30, Supporting Information). As can be seen that both disk and ring currents display slight change during the entire test period, meaning high 2e^−^ ORR stability. To manifest the applicability of Ni–O–C for H_2_O_2_ production through electrocatalytic 2e^−^ ORR process, the electrochemical experiments were also performed using Ni–O–C coated carbon paper electrode at different potentials in a three‐electrode configured H‐type electrochemical cell with continuous O_2_ bubbling (Figure S31, Supporting Information). The H_2_O_2_ amount produced was determined by the Ce^4+^/Ce^3+^ colorimetric method (Figure S32a–c, Supporting Information).^[^ ^14^, ^15^ ^]^ As shown in Figure S32d (Supporting Information), a high H_2_O_2_ yield rate of 1.08 ± 0.03 mol g_cat._^−1^ h^−1^ with the largest faradaic efficiency (FE) of 92.5 ± 3.3% can be attained at 0.4 V (vs RHE), outperforming most of recently reported 2e^−^ ORR electrocatalysts (Table S1, Supporting Information). The above experimental results demonstrated superior 2e^−^ ORR activity of Ni–O–C for H_2_O_2_ synthesis. After 12 h of reaction at 0.4 V (vs RHE) in O_2_‐saturated 0.1 m KOH electrolyte, the produced H_2_O_2_ concentration is around 1.1 mmol in cathodic compartment in such three‐electrode configured H‐type electrochemical cell. When 0.5 mmol benzene was added to the electrolyte containing 1.1 mmol H_2_O_2_ with an applied anodic potential of 1.5 V (vs RHE) for 2 h of reaction, the conversion of benzene was found to be 54.5% with a FE of 79.1% and 100% phenol selectivity (Figure S33, Supporting Information). The above results verified the feasibility of coupling electrocatalytic synthesis of H_2_O_2_ and benzene oxidation for high‐value phenol production at ambient conditions.

### 2.5 Two‐Electrode System

The aforementioned electrochemical experiments have confirmed the Ni–O–C with bifunctionality of electrocatalytic benzene oxidation to phenol and 2e^−^ ORR to H_2_O_2_. As a proof of concept experiment, a two‐electrode system composed of Ni–O–C coated on carbon paper concurrently as the anode and cathode in a single cell was established for evaluation of the electrocatalytic benzene oxidation performance. As illustrated in Figure S34 (Supporting Information), all electrochemical measurements were performed at a current density of 10 mA cm^−2^ in O_2_‐saturated 0.1 m KOH electrolyte containing 0.5 mmol benzene. After 2 h of reaction, the conversion of benzene reached 33.4 ± 2.2% with 100% selectivity of phenol (Figure S35, Supporting Information). This can be speculated that under electrocatalysis conditions, 2e^−^ ORR happened on Ni–O–C cathode generated H_2_O_2_ that was further oxidized on Ni–O–C anode to form the active oxygen species for benzene oxidation to phenol. To verify this, a control experiment was conducted under the same experimental conditions except platinum mesh instead of Ni–O–C as the cathode. The result indicated that no phenol product was detected after 2 h of reaction (Figure S35, Supporting Information). As we know, platinum is a very outstanding electrocatalyst for 4e^−^ ORR to H_2_O but not for 2e^−^ ORR to H_2_O_2_,^[^ ^32^ ^]^ therefore it is reasonable no phenol product obtained owing to inexistence of H_2_O_2_ in the reaction system, further indicating the significant role of H_2_O_2_ in electrocatalytic benzene oxidation to phenol. Furthermore, we have estimated the energy efficiency (59.3%) for two‐electrode system at a current density of 10 mA cm^−2^ (Table S5, Supporting Information).

### 2.6 Mechanistic Studies

**Figure 4:**
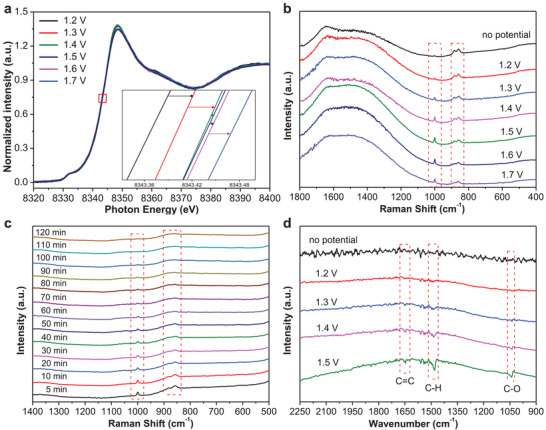
Mechanistic studies. a) XANES spectra of the Ni *K* edge of Ni–O–C under various potentials for Ni–O–C during electrocatalytic oxidation of benzene to phenol. b) Operando Raman spectroscopy measurements under various potentials for Ni–O–C during electrocatalytic oxidation of benzene to phenol. c) Operando Raman spectroscopy measurements of Ni–O–C during electrocatalytic oxidation of benzene to phenol at 1.5 V (vs RHE) for 2 h. d) Operando SR‐FTIR spectroscopy measurements under various potentials for Ni–O–C during electrocatalytic oxidation of benzene to phenol.

To unveil the electrocatalytic active mechanisms of benzene oxidation to phenol, a series of characterizations measurements were subsequently performed. **Figure** **4**a presents the XANES spectra at Ni K edge of Ni–O–C recorded at different applied potentials. The energy position of the absorption edge gradually shifted to higher energy with the increased potential, suggesting that the Ni species in Ni–O–C tended to higher valence at more positive potential with more superior reactivity.^[^ ^33^, ^34^ ^]^ For electrocatalytic benzene oxidation to phenol in this work, a suitable anodic potential is critically important because higher anodic potentials could be beneficial for the competitive oxygen evolution reaction (OER), consistent with the potential‐dependence experimental results (Figure 3b). In addition, the operando Raman measurements were conducted to study the electrochemical oxidation of benzene on Ni–O–C in 0.1 m KOH electrolyte over the potential range from 1.2 to 1.7 V (vs RHE) (Figure S36, Supporting Information). As shown in Figure 4b, when no potential was applied, several Raman peaks appeared at around 858.8, 880.7, and 1000 cm^−1^, respectively. The peaks at around 858.8 and 880.7 cm^−1^ were attributed to the OH bending mode of H_2_O_2_, and the weak peak situated at around 1000 cm^−1^ was assigned to the vibration of benzene ring.^[^ ^11^, ^35^ ^]^ When the anodic potential was applied, the Raman peak intensity at around 1000 cm^−1^ enhanced obviously, due to the enhanced vibration and activation of benzene ring on Ni–O–C surface, meaning its effective activation with the applied potential. We further investigated the electrochemical oxidation of benzene to phenol at 1.5 V (vs RHE) for 2 h by the operando Raman measurements. The Raman intensity of the peak at around 1000 cm^−1^ belonging to the vibration of benzene ring decreased significantly from 5 to 100 min and completely disappeared by 110 min (Figure 4c). It was also found that the Raman intensity of the peaks belonging to H_2_O_2_ at around 858.8 and 880.7 cm^−1^ gradually decreased from 5 to 120 min (Figure 4c). These results suggested that the reaction precursor of benzene is gradually oxidized with time under the given electrocatalytic conditions. Furthermore, the advanced operando synchrotron radiation FTIR (SR‐FTIR) measurements^[^ ^36^ ^]^ were also conducted on Ni–O–C to validate the active mechanisms of electrochemical oxidation of benzene to phenol. Figure S37 (Supporting Information) shows the experimental set‐up for the SR‐FTIR measurements. The infrared signals were collected during the positive scan from 1.2 to 1.5 V (vs RHE) (Figure 4d), the infrared bands were probed at 1648 and 1478 cm^−1^, which were assignable to the C=C stretching mode and C–H bending mode in benzene ring.^[^ ^37^ ^]^ Apart from the infrared bands of C=C and C–H, the stretching mode of C–O can be also observed at 1047 cm^−1^ with enhanced infrared band intensity at higher applied potential, implying much higher hydroxylation efficiency of benzene.^[^ ^37^ ^]^ Evidenced by the operando SR‐FTIR measurements, the selective electrocatalytic oxidation of aromatic inactive C–H bonds was successfully realized for phenol production in this work.

**Figure 5:**
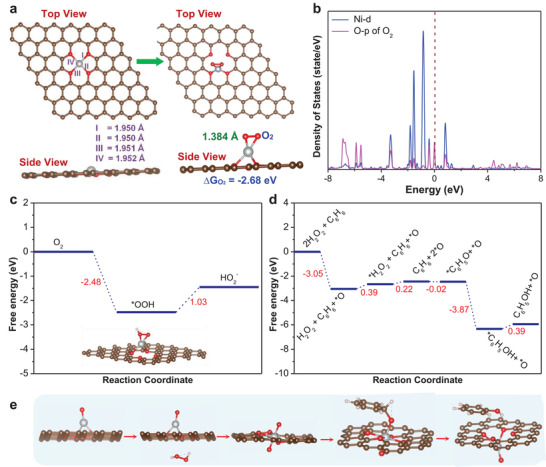
Theoretical analysis. a) DFT optimized configurations of Ni–(O–C_2_)_4_ and O_2_ adsorption on Ni–(O–C_2_)_4_. b) The computed projected density of states (PDOS) of Ni–O–C after O_2_ adsorption. The Fermi level was set to be as denoted by the wine dashed line. c) Gibbs free energy diagram of the 2e^−^ ORR pathway and DFT calculated intermediate (*OOH) structure on Ni–(O–C_2_)_4_. d) Gibbs free energy diagram of the electrocatalytic H_2_O_2_ activation and benzene oxidation pathway and e) DFT calculated intermediates structures on Ni–(O–C_2_)_4_ (Brown: C, red: O, White: Ni, Silver: H).

In this work, the experimentally identified Ni–SA site configuration was further used to construct the structural model for the density functional theory (DFT) calculations. The binding energy was firstly calculated for evaluating the stability of Ni–(O–C_2_)_4_ configuration. As calculated, the negative binding energy value (−1.81 eV) indicated that Ni is not prone to aggregation. **Figure** **5**a shows the DFT optimized Ni–(O–C_2_)_4_ configuration on graphitic carbon with four Ni–O bond lengths of 1.950, 1.950, 1.951, and 1.952 Å, respectively, basically consistent with the EXAFS data (2.05 Å). In addition, AIMD simulations were further performed to assess the thermal stability of Ni–(O–C_2_)_4_.^[^ ^38^ ^]^ As illustrated in Figure S38 (Supporting Information), the energy and temperature fluctuate in small ranges without significant changes, further demonstrating that the formation of Ni–(O–C_2_)_4_ configuration is thermodynamically feasible. Based on the optimized Ni–(O–C_2_)_4_ site, we firstly studied the interaction between O_2_ and Ni–O–C. The O_2_ adsorption free energy (Δ*G*_O2_) was −2.68 eV via a side‐on configuration (Figure 5a), and the O–O distance was elongated from 1.208 Å (free O_2_ molecule) to 1.384 Å (adsorbed O_2_ molecule), indicating that the adsorbed O_2_ (^*^O_2_) on Ni–(O–C_2_)_4_ site was effectively activated. Furthermore, the projected density of states (PDOS) results (Figure 5b) demonstrated that there was an obvious hybridization between the O‐2p orbitals and Ni‐3d orbitals after O_2_ adsorption, especially near the Fermi level, reflecting the strong chemical adsorption of O_2_, which was beneficial for the subsequent hydrogenation process.^[^ ^39^ ^]^ To evaluate the 2e^−^ ORR catalytic activity of Ni–(O–C_2_)_4_, we computed full reaction pathways. In the process of 2e^−^ ORR to H_2_O_2_ in alkaline media, the catalytic activity can be determined by preventing the O–O bond dissociation.^[^ ^40^ ^]^ As shown in Figure 5c, the reduction energy of ^*^OOH to HO_2_^−^ is uphill and the value is 1.03 eV, indicating the Ni–(O–C_2_)_4_ configuration was prone to reduce O_2_ to H_2_O_2_.

Additionally, we also carried out the DFT calculations to investigate the electrocatalytic active mechanisms of benzene oxidation to phenol with the help of H_2_O_2_ on Ni–(O–C_2_)_4_ site. The free energy profile and reaction pathway of benzene oxidation in the presence of H_2_O_2_ on Ni–(O–C_2_)_4_ are shown in Figure 5d and Figure S39 (Supporting Information), and the optimized intermediate structure corresponding to each reaction step is illustrated in Figure 5e. Previously, Zhou et al. reported that first the interaction of catalyst with H_2_O_2_ initially dissociates OOH* species via a redox mechanism, and then the unstable OOH* radical attacks the benzene ring and easily forms the active oxygen species (O*) to oxidize benzene to phenol.^[^ ^28^ ^]^ Based on this, the calculations results revealed that an H_2_O_2_ molecule firstly dissociated on the confined Ni–(O–C_2_)_4_, which was an exothermic process with the △*G* of −3.05 eV by forming Ni=O* intermediate to release one H_2_O molecule,^[^ ^2^, ^28^ ^]^ which is much lower than Ni–N_2_O_2_ (−1.55 eV) and Ni–N_4_ (0.57 eV) (Figure S40 and S41, Supporting Information). Therefore, Ni–(O–C_2_)_4_ sites would be more beneficial for the H_2_O_2_‐assisted benzene oxidation process. Subsequently, the second H_2_O_2_ adsorbed on the other side of Ni atom needing an energy inject of 0.39 eV, finally dissociated forming the *O=Ni=O* center with a slight energy increase of 0.22 eV. Benzene molecule attached to the active *O species of *O=Ni=O* and formed the C–O bond. This was a spontaneous process accompanied by a downhill energy barrier of −0.24 eV. The adjacent H atom from C of benzene subsequently transferred to O of C–O, achieving the transformation of benzene to phenol with a Gibbs free energy change of −3.87 eV. After the phenol desorption from Ni active site, the Ni=O* and *O=Ni=O* sites can regenerate during the reaction. We further conducted the DFT calculations for the path energy of the catechol side‐product product (Figure S42, Supporting Information). The O species of the O=Ni=O center was active for the adsorption of the phenol via the formation of a C–O bond with an energy barrier of −0.12 eV. However, the phenol adsorbed on the O=Ni=O site can transform to catechol via the transfer of one adjacent H atom from C to O with a high barrier of 2.87 eV, which is much higher than phenol (−3.87 eV). Therefore, catechol is more difficult to form than phenol. The above results unveiled that the Ni–(O–C_2_)_4_ active site is thermodynamically favorable for the hydroxylation of benzene.

## 3 Conclusion

In summary, an oxygen‐coordinated nickel single‐atom catalyst (Ni–O–C) was successfully fabricated through an adsorption‐regulated synthetic strategy using bacterial cellulose (BC) as the adsorption substrate. The as‐synthesized Ni–O–C exhibited superior bifunctional electrocatalytic activities toward the 2e^−^ ORR to H_2_O_2_ and H_2_O_2_‐assisted benzene oxidation to phenol. The Ni–O–C can afford a high H_2_O_2_ yield rate of 1.08 ± 0.03 mol g_cat._^−1^ h^−1^ and a FE of 92.5 ± 3.3% at 0.4 V (vs RHE) in 0.1 m KOH, and a benzene conversion as high as 96.4 ± 3.6% with a phenol selectivity of 100% and a FE of 80.2 ± 3.2% with the help of H_2_O_2_ in 0.1 m KOH at 1.5 V (vs RHE). The experimental and theoretical studies confirmed that the Ni–(O–C_2_)_4_ sites in Ni–O–C were the catalytic active centers for 2e^−^ ORR to H_2_O_2_ and H_2_O_2_‐assisted benzene oxidation to phenol. A two‐electrode proof of concept experiment in this work verified the feasibility of coupling electrocatalytic synthesis of H_2_O_2_ and benzene oxidation for high‐value phenol production at ambient conditions.

## Conflict of Interest

The authors declare no conflict of interest.

## Supporting information

Supporting Information

## Data Availability

The data that support the findings of this study are available in the supplementary material of this article.
