# Supplementary material for: Hydrogen Peroxide Assisted Electrooxidation of Benzene to Phenol over Bifunctional Ni–(O–C2)4 Sites
Source: Adv Sci (Weinh). 2022 Oct 30;9(35):2204043. doi: 10.1002/advs.202204043 (PMC9762286; doi:10.1002/advs.202204043)
Supplement: Supplementary file 1 — Supporting Information [file ADVS-9-2204043-s001.pdf]

ADVANCED SCIENCE
Open Access

Supporting Information

for *Adv. Sci.*, DOI 10.1002/advs.202204043

Hydrogen Peroxide Assisted Electrooxidation of Benzene to Phenol over Bifunctional Ni–(O–C_2)$_4$ Sites

Shengbo Zhang, Meng Jin, Hui Xu, Wenyi Li, Yixing Ye, Tongfei Shi, Hongjian Zhou, Chun Chen, Guozhong Wang, Yunxia Zhang, Yue Lin, Lirong Zheng*, Haimin Zhang* and Huijun Zhao*

Supporting Information

Hydrogen peroxide assisted electrooxidation of benzene to phenol over bifunctional Ni-(O-C_2)$_4$ sites

Shengbo Zhang, Meng Jin, Hui Xu, Wenyi Li, Yixing Ye, Tongfei Shi, Hongjian Zhou, Chun Chen, Guozhong Wang, Yunxia Zhang, Yue Lin, Lirong Zheng*, Haimin Zhang*, Huijun Zhao*

Dr. S. Zhang, Dr. M. Jin, H. Xu, Dr. W. Li, Dr. Y. Ye, Dr. T. Shi, Dr. H. Zhou, Dr. C. Chen, Prof. G. Wang, Prof. Y. Zhang, Prof. H. Zhang
Key Laboratory of Materials Physics, Centre for Environmental and Energy Nanomaterials, Anhui Key Laboratory of Nanomaterials and Nanotechnology, CAS Center for Excellence in Nanoscience, Institute of Solid State Physics, HFIPS, Chinese Academy of Sciences, Hefei 230031, China
University of Science and Technology of China, Hefei 230026, China
E-mail: zhanghm@issp.ac.cn

Dr. Y. Lin
Hefei National Research Center for Physical Sciences at the Microscale, University of Science and Technology of China, Hefei 230026, China
E-mail: linyue@ustc.edu.cn

Prof. L. Zheng
Beijing Synchrotron Radiation Facility, Institute of High Energy Physics, Chinese Academy of Sciences, 19B Yuquan Road, Beijing 100049, China
E-mail: zhenglr@ihep.ac.cn

Prof. H. Zhao
Centre for Catalysis and Clean Energy, Griffith University, Gold Coast Campus, QLD 4222, Australia

S. Zhang, M. Jin and H. Xu contributed equally.

Experimental Section

Methods

Reagents and materials. Bacterial cellulose (BC) pellicle was obtained from Guilin Qihong Technology Co., Ltd., China. $Ni(NO_3)_2{\cdot}6H_2O$ (98.5%), HCl (36.0-38.0%), $Ce(SO_4)_2{\cdot}4H_2O$ (98.0%), ethyl acetate (99.8%), H_2O_2 (30%) and C_2H_5OH (75.0%) were purchased from Sinopharm Chemical Reagent Co., Ltd. KOH (99.0%), benzene (99.9%), methylbenzene (99.9%), nitrobenzene (99.0%) and bromobenzene (99.0%) were purchased from Aladdin. (All solutions were prepared using deionized water (Millipore Corp., 18.2 MΩ cm). Commercial carbon paper was purchased from Shanghai Hesen Electric Co. Ltd.

Fabrication of Ni-O-C. BC pellicle was frozen by liquid nitrogen and freeze-dried in a bulk tray dryer at a sublimating temperature of -75 °C and a pressure of 0.01 mbar for 48 h. To remove organic contaminations, the freeze-dried BC was dispersed in 200 mL piranha solution under constant stirring at room temperature for 6 h, thoroughly washed with deionized water and freeze-dried. The pre-treated BC was used as the adsorbent to controllably impregnate Ni^{2+}. For Ni^{2+}-BC, 1.0 g pre-treated BC was dispersed in 400 mL of 240 mmol L^{-1} of Ni^{2+} solution at room temperature for 6 h to complete the adsorption. The obtained Ni^{2+}-BC was adequately washed with deionized water, freeze-dried and carbonised in a tubular furnace under an Ar atmosphere. The sample was firstly heated to 360 °C with a heating rate of 2 °C min^{-1} and kept for 2 h, then heated to 700 °C with a heating rate of 5 °C min^{-1} and kept for 3 h to carbothermally reduce the adsorbed Ni^{2+} on BC to metallic Ni NPs and simultaneously carbonise BC into graphitic carbon (CBC). The resultant Ni-CBC were adequately washed with the Millipore water and ethanol, dried at 60 °C under vacuum for 6 h, then subjected to a refluxing acid-etching process using 4.0 M HCl at 120 ^{o}C for 4 h to remove metallic Ni NPs. The acid etched Ni-CBC was thoroughly washed by deionised water and ethanol, and dried at 60 °C under vacuum for 12 h to obtain Ni-O-C.

Characterization. XRD patterns were acquired using Philips X'pert PRO with Cu Ka radiation (λ = 1.5418 Å) at 40 kV and 40 mA. FT-IR measurements were conducted by a Nicolet Nexus FT-IR

spectrometer. Raman spectra were recorded by a Renishaw Micro-Raman Spectroscopy (Renishaw in Via Reflex) with 532 nm excitation laser. SEM images were obtained using SU8020 (Hitachi, Japan). TEM images were obtained using JEMARM 200F. HAADF-STEM measurements and EDX spectroscopy were performed on a JEM-ARM200F. XPS spectra were obtained using an ESCALAB 250 X-ray photoelectron spectrometer (Thermo, America). Nitrogen adsorption-desorption isotherms were measured using Autosorb-iQ-Cx. The synchrotron-based X-ray absorption measurements were performed at the 1W1B station of Beijing Synchrotron Radiation Facility, China. Ni^{2+} and metallic Ni contents were quantitatively determined by ICP-AES (ICP-6300, Thermo Fisher Scientific). For the *operando* Raman tests, the samples were recorded on a RXN1-785 Raman spectrometer (Analytik Jena AG, excited wavelength of 785 nm) connected with CHI 660E electrochemical workstation (Figure S36). The operando SR-FTIR measurements were conducted at the infrared beamline BL01B of the National Synchrotron Radiation Laboratory through a homemade top-plate cell-reflection infrared set-up with a ZnSe crystal as the infrared transmission window (cut-off energy of ~625 cm^{-1}). This end station was equipped with an FTIR spectrometer (Bruker 70 v/s) with a KBr beam splitter and various detectors (herein, a liquid-nitrogen-cooled mercury cadmium telluride detector was used) coupled with an infrared microscope (Bruker Hyperion 2000) with an × 15 objective. The catalyst electrode was tightly pressed against the ZnSe crystal window with a micrometer-scale gap to reduce the loss of infrared light. To ensure the quality of the obtained SR-FTIR spectra, the apparatus adopted a reflection mode with a vertical incidence of infrared light. Each infrared absorption spectrum was acquired by averaging 128 scans at a resolution of 4 cm^{-1}. The background spectrum of the catalyst electrode was acquired at an open-circuit voltage before each systemic measurement, and the measured potential ranges of the electrocatalytic oxidation reaction were 1.2 to 1.5 V *vs.* RHE with an interval of 0.1 V. The operando electrochemical set-up is shown in Figure S37.

Electrochemical measurements. All electrochemical measurements were performed on a CHI 760E electrochemical workstation (CH Instrumental Corporation, Shanghai, China) under ambient

conditions using a Nafion 211 proton exchange membrane separated two-compartment H-type electrochemical cell accommodated 50 mL of 0.1 M KOH electrolyte in each compartment and a three-electrode electrochemical system with a Ni-O-C based working electrode, an Ag/AgCl (Saturated KCl) reference electrode and a Pt mesh counter electrode. Before use, the Nafion 211 membrane was treated by successive heating at 80 ^{o}C in H_2O_2 (5.0 wt.%) aqueous solution for 1 h and in deionised water for another 1 h. The working electrode was prepared as follows: 2.0 mg of the targeted electrocatalyst was firstly dispersed in 95 μL of absolute ethanol and 5 μL of Nafion solution (5.0 wt.%) unnder sonication for 30 min to form a homogeneous ink. 100 μL ink was loaded onto a carbon paper electrode (1×1 cm^2, equivalent to 2.0 mg cm^{-2}) and dried under ambient conditions for 40 min before use. Before the reaction, Ar gas was persistently bubbled into the electrolyte to eliminate O_2 interference. 0.5 mmol benzene + 5.0 mmol H_2O_2 were then added to the anode cell and stirred continuously during the electrocatalytic process. After the reaction, we further washed the electrode with deionized water and ethanol to completely remove the potential adsorption of benzene and its oxygenated products on the surfacethe. Then the washed solution and the entire reaction solution in the anode compartment was collected, adjusted to pH = 5.0, concentrated by rotary evaporation to remove the solvents, then extracted with ethyl acetate and dried over anhydrous sodium sulfate. The obtained products were quantitatively analyzed by gas chromatography (GC) measurement. In ^{1}H NMR experiments (Bruker Avance-400 MHZ), d6-DMSO (as internal standard) was used for analysis.

The electrochemical ORR measurements were performed on a CHI 760E electrochemical workstation with a three-electrode cell and a rotating ring-disk electrode setup (RRDE-3A, ALS Co., Ltd). A RRDE electrode with a glassy carbon electrode (O.D. 4.0 mm) and a platinum ring electrode (I.D. 5.0 mm, O.D. 7 mm) was used as the working electrode. Before measurement, the electrode was polished with 0.3 and 0.05 μm alumina suspensions on a polishing cloth and rinsed with Millipore water. A Ag/AgCl electrode and Pt wire were used as the reference and counter electrode, respectively. Three electrolytes with 0.1 M KOH was used. The catalyst loading amount

was 0.025 mg cm^{-2}. The H_2O_2 selectivity was obtained from polarization curves in O_2-saturated condition at a scan rate of 10 mV s^{-1} at 1600 rpm and the potential of the Pt ring electrode were held at 1.2 V to oxidize the H_2O_2 generated on disk electrode.

The H_2O_2 selectivity, the calculation method of electron transfer number (*n*) on the RRDE and Koutecky-Levich (K-L) equations based on the previously reported literatures.[1] Electrocatalytic H_2O_2 production was conducted in a two-compartment H-cell with Nafion 211 membrane as separator. Each compartment was filled with 40 mL of electrolyte (0.1 M KOH). We used the carbon paper (1×1 cm^2) as the working electrode with a catalyst loading amount of 0.1 mg cm^{-2}, Ag/AgCl and Pt mesh as the reference electrode and counter electrode, respectively. Before the measurement, the electrolyte in the cathode compartment was purged with O_2 for at least 30 min to reach saturated and stirred vigorously to facilitate the mass transport of O_2.

In this work, all measured potentials *vs.* Ag/AgCl were converted to the potentials *vs.* RHE (E_{RHE}) according to the following equation:

$$E_{\mathrm{RHE}} = E_{\mathrm{Ag/AgCl}} + 0.059\mathrm{pH} + E^{\circ}{}_{\mathrm{Ag/AgCl}}$$

where, $E_{Ag/AgCl}$ is the equilibrium potential under standard conditions, $E^{o}{}_{Ag/AgCl}$ = 0.1967 V *vs.* RHE at 25 ^{o}C.

Calculation of selectivity toward phenol. The selectivity toward phenol, $S_{C_6H_5OH}$ was determined using the following equation:

$$S_{\mathrm{C_6H_5OH}} = \frac{\mathrm{Observed\ \ C_6H_5OH\ (mmol\,)}}{\mathrm{Total\ \ product\ \ (mmol\,)}} \times 100\%$$

Calculation of H_2O_2 concentration. The H_2O_2 concentration in the electrolyte was quantified by ceric sulfate titration following the reaction:

$$2Ce^{4+} + H_2O_2 \rightarrow 2Ce^{3+} + 2H^{+} + O_2$$

where, the yellow-colored Ce^{4+} was reduced by H_2O_2 to colorless Ce^{3+} and can be measured by ultraviolet-visible spectroscopy. A series of standard $Ce(SO_4)_2$ solution was prepared by dissolving $Ce(SO_4)_2$ in 0.5 M H_2SO_4, then we obtained the calibration curves between the absorbance and

concentration of Ce^{4+} performed on spectrophotometer at 317 nm. Thus, the concentration of H_2O_2 can be calculated based on the absorbance before and after reaction.

Calculation of FE. The FEs of phenol and H_2O_2 were calculated by the following equation:[1,2]

$$\text{FE (\%)} = \frac{2 \times n_{C_6H_5OH}(\text{mol}) \times F\ (\text{C mol}^{-1})}{Q\ (\text{C})} \times 100\%$$

$$\text{FE (\%)} = \frac{2 \times n_{H_2O_2}(\text{mol}) \times F\ (\text{C mol}^{-1})}{Q\ (\text{C})} \times 100\%$$

where, F is the Faradaic constant (96485 C mol^{-1}) and Q is the total charge transferred during the electrocatalysis period.

Theoretical calculations. All the spin-polarized DFT calculations were performed using the Vienna Ab-initio Simulation Package (VASP),[3] and the generalized gradient approximation (GGA) with the Perdew-Burke-Ernzerhof (PBE) exchange-correlation functional was used with the projector augmented wave method.[4] A kinetic energy cutoff of 500 eV was used for plane wave expansion, the convergence threshold was set as 10^{-5} eV in energy and 0.02 eV $Å^{-1}$ in force. The p(6×6) Ni-(O-$C_2)_4$ supercell was constructed. The Brillouin zone was sampled with a 2 × 1 × 1 Monkhorst-Pack k-points grid for geometric optimization of slab models, respectively. The vacuum distance was set to 20 Å to minimize the artificial interactions of the interlayer.[5] During the total calculations, the symmetry was switched off and the dipolar correction was also included. DFT-D3 method was used for the dispersion correction.[6] The change of the Gibbs free energy of each step was calculated as followings equations:[7]

$$\Delta G = \Delta E + \Delta E_{ZPE} - T\Delta S$$

where, ΔE is the energy change between the reactant and product species, ΔE_{ZPE} is the difference in zero point energy, T is the temperature (T = 298.15 K) and ΔS is the entropy difference. The binding energy was calculated as follows:

$$E = E_{total} - EM - E_{support}$$

where, E_{total} represents the optimized total energy of support complex, EM and Esupport represent the total energy of Fe atom and support, respectively. Bulk Ni was used for the reference to calculate the total energy of Ni atom. Ab initio molecular dynamics simulations (AIMD) were employed to evaluate the thermodynamic stability of the materials. AIMD simulations for 10 ps within the NVT ensemble using a time step of 2 fs at 300 K. The VASPKIT code was used for the post-processing of the DFT calculated data.[8]

Table S1. 2e$^-$ ORR performance of the reported ORR electrocatalysts and Ni-O-C in this work.

**References**	**Catalyst**	**Electrolyte**	**Onset Potential (V *vs.* RHE)**	**Selectivity (%)**	**Production Rate**
[9]	Co_1-NG(O)	0.1 M KOH	~0.83	82	418 ± 19 mmol g_{cat}^{-1} h^{-1}
[10]	Co-POC-O	0.1 M KOH	0.84	~84	813 mg L^{-1} h^{-1}
[11]	Co_{SA}-N-CNTs	0.5 M H_2SO_4	0.7	95	974 ± 25 mmol g_{cat}^{-1} h^{-1}
[12]	Co_1@GO	0.1 M KOH	0.91	81.4	1.0 mg cm^{-2} h^{-1}
[13]	CoNOC	0.1 M $HClO_4$	0.57	95	590 mmol g_{cat}^{-1} h^{-1}
[14]	ZnO_3C	0.1 M KOH	~0.75	80	350 mmol g_{cat}^{-1} h^{-1}
[15]	Pt/TiN	0.1 M $HClO_4$	~0.6	65	-
[16]	Pt/TiC	0.1 M $HClO_4$	~0.6	68	-
[17]	h-Pt_1-CuS_x	0.1 M $HClO_4$	~0.7	96	546 ± 30 mmol g_{cat}^{-1} h^{-1}
[18]	O-C(Al)	0.1 M NaOH	0.822	95	867 mg L^{-1} h^{-1}
[19]	W_1/NO-C	0.1 M KOH	0.815	90	1.23 mol g_{cat}^{-1} h^{-1}
[20]	Mo_1/OSG-H	0.1 M KOH	0.78	95	-
[21]	O-$CoSe_2$	0.05 M H_2SO_4	~0.75	76	15.18 mmol g_{cat}^{-1} h^{-1}
[22]	CoS_2	0.05 M H_2SO_4	0.69	~70	38.56 mmol g_{cat}^{-1} h^{-1}
[23]	$CoSe_2$@NCNTs	0.1 M $HClO_4$	0.68	93.2	172 mg L^{-1} h^{-1}
[24]	sc-$CoSe_2$	0.5 M H_2SO_4	~0.7	95	30.60 mg cm^{-2} h^{-1}
[25]	$Ni_2Mo_6S_8$	0.1 M KOH	0.75	90	90 mmol g_{cat}^{-1} h^{-1}
[26]	Ni MOF NSs-6	0.1 M KOH	~0.75	98	80 mmol g_{cat}^{-1} h^{-1}
[27]	MnO/N@NCs-50	0.1 M $HClO_4$	0.78	74	20 μmol h^{-1} cm^{-2}
[28]	O-BC-2-650	0.1 M KOH	~0.8	98	412.8 mmol g_{cat}^{-1} h^{-1}
[29]	$OCNS_{900}$	0.1 M KOH	0.825	90	770 mmol g_{cat}^{-1} h^{-1}
[30]	oxo-G/$NH_3 \cdot H_2O$	0.1 M KOH	~0.8	82	224.8 mmol g_{cat}^{-1} h^{-1}
[31]	HPCS-S	0.1 M KOH	0.77	70	183.99 mmol g_{cat}^{-1} h^{-1}
[32]	rGO-PEI	0.1 M KOH	0.8	90.7	106.4 mmol g_{cat}^{-1} h^{-1}
This work	Ni-O-C	0.1 M KOH	0.4	88.1	1.08 ± 0.03 mol $g_{cat.}^{-1}$ h^{-1}

Table S2. Structural parameters extracted from the Ni *K*-edge EXAFS fitting data of Ni-O-C before and after 8 test cycles.

**Sample**	**Scattering Pair**	**CN**	**R (Å)**	**$\sigma^2(10^{-3}Å^2)$**	**ΔE_0 (eV)**
Ni-O-C (As synthesised)	Ni-O	4.0	2.05	4.6	-0.9
Ni-O-C (After 8 test cycles)	Ni-O	4.0	2.05	4.7	-0.7

CN is the coordination number; R is interatomic distance (the bond length between central atoms and surrounding coordination atoms); σ^2 is Debye-Waller factor (a measure of thermal and static disorder); ΔE_0 is edge-energy shift (the difference between the zero kinetic energy value of the sample and that of the theoretical model).

Table S3. Benzene oxdation performance of the reported catalysts and Ni-O-C in this work.

Reference	Catalyst	Conditions	Benzene conversion (%)	H_2O_2 consumption efficiency (%)	Phenol selectivity (%)
		**Thermal-catalysts**			
[33]	Cu_1-N_3O_1 (Cu-N/O)	Phenol (300 μL) H_2O_2 (5 mL), CH_3CN (60 °C), 12 h	70.9	1.9	94.0
[34]	Cu_1/GDY (Cu-C)	Phenol (300 μL) H_2O_2 (5 mL), CH_3CN (60 °C), 12 h	86	1.9	96
[35]	SA Fe/C (Fe-N bond)	Phenol (1 mL) H_2O_2 (16 mL), CH_3CN (60 °C), 12 h	84.8	2.0	99
[36]	Cu_1/NC-1000 (Cu-N bond)	Phenol (300 μL) H_2O_2 (5 mL), CH_3CN (60 °C), 12 h	82	1.9	96
[37]	CuN_3-S-C (Cu-N/S bond)	Phenol (200 μL) H_2O_2 (3 mL), CH_3CN (70 °C), 24 h	42.3	2.1	93.4
[38]	Fe-NxCy (Fe-N bond)	Phenol (400 μL) H_2O_2 (6 mL), CH_3CN (80 °C), 12 h	78.4	2.1	100
[39]	Co-ISA/CNS (Co-N bond)	Phenol (400 μL) H_2O_2 (6 mL), CH_3CN (80 °C), 12 h	68	2.1	61
[40]	Cu-SA/HCNS (Cu-N bond)	Phenol (400 μL) H_2O_2 (6 mL), CH_3CN (60 °C), 12 h	86.0	2.1	96.7
[41]	SA-Fe/CN (Fe-N bond)	Phenol (400 μL) H_2O_2 (6 mL), CH_3CN (60 °C), 24 h	45	2.1	94
[42]	FeN_4/GN-2.7 (Fe-N bond)	Phenol (400 μL) H_2O_2 (6 mL), CH_3CN (25 °C), 24 h	23.4	2.1	18.7
		**Photocatalyst**			
[43]	Zn_xTi-LDH	H_2O	5.7	-	81.12
		**Electrocatalyst**			
[44]	V_2O_5	H_2O, 50 °C	41.7	-	100
[45]	Pt	TFA, 2,6-lutidine, MeCN, current: 61-173 mA	96	-	100
This work	Ni-O-C (Ni-O bond)	H_2O_2, H_2O	96.4 ± 3.6	10	100

Table S4. Energy efficiency results obtained from applied potentials*.

**Potential (V *vs.* RHE)**	**$E_{Cathode}$ (V *vs.* RHE)**	**E_{Anode} (V *vs.* RHE)**	**ΔE (Applied)† (V *vs.* RHE)**	**Energy efficiency* (%)**
1.2 V	0.43	1.2	0.77	156
1.3 V	0.01	1.3	1.29	100
1.4 V	-0.11	1.4	1.51	92.7
1.5 V	-0.21	1.5	1.71	87.7
1.6 V	-0.35	1.6	1.95	82.1
1.7 V	-0.54	1.7	2.24	75.9

†ΔE (Applied) = E(cathode)-E(anode).
*Energy efficiency (%) = E_{Anode}/ΔE (Applied)

Table S5. Energy efficiency result of two-electrode system*.

**Current/Potential (mA/V)**	**ΔE (Applied)† (V)**	**Energy efficiency* (%)**
2.41	2.68	59.3

†ΔE (Applied) = E(cathode)-E(anode).

*Energy efficiency (%) = (Electric potential-0.822)/ΔE (Applied), the onset potential for ORR to H_2O_2 can be 0.822 V (*vs*. RHE).[46]

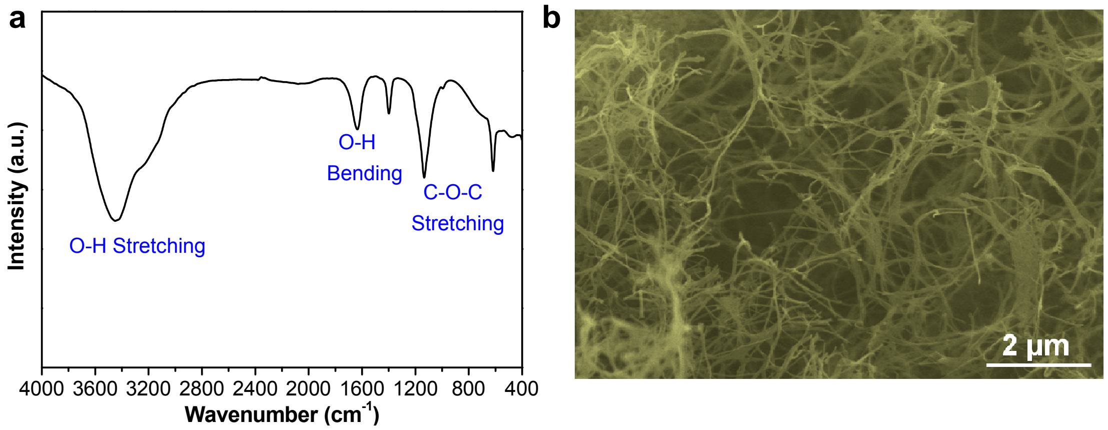

Figure S1. (**a**) FT-IR spectrum and (**b**) SEM image of pre-treated BC.

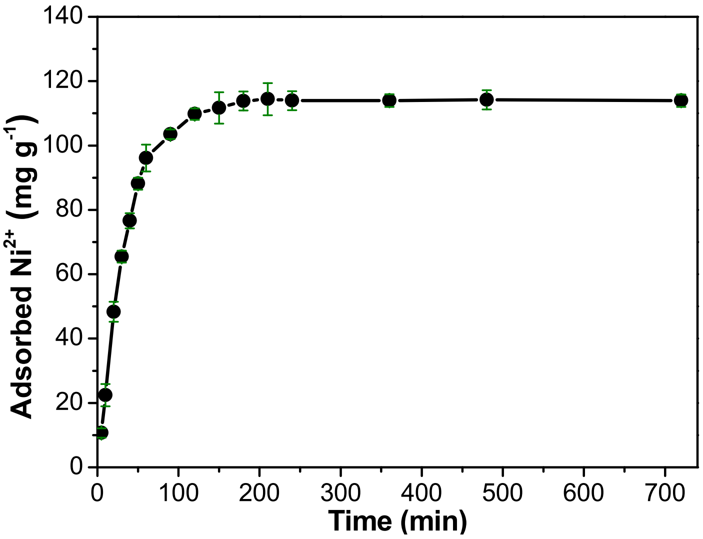

Figure S2. Adsorption of Ni^{2+} on pre-treated BC at 25 °C. The concentration of Ni^{2+} in adsorption solution: 240 mmol L^{-1}; Adsorption solution volume: 400 mL; Amount of BC: 1.0 g; q_e: impregnated Ni^{2+} amount on BC under equilibrium condition determined by the ICP-AES.

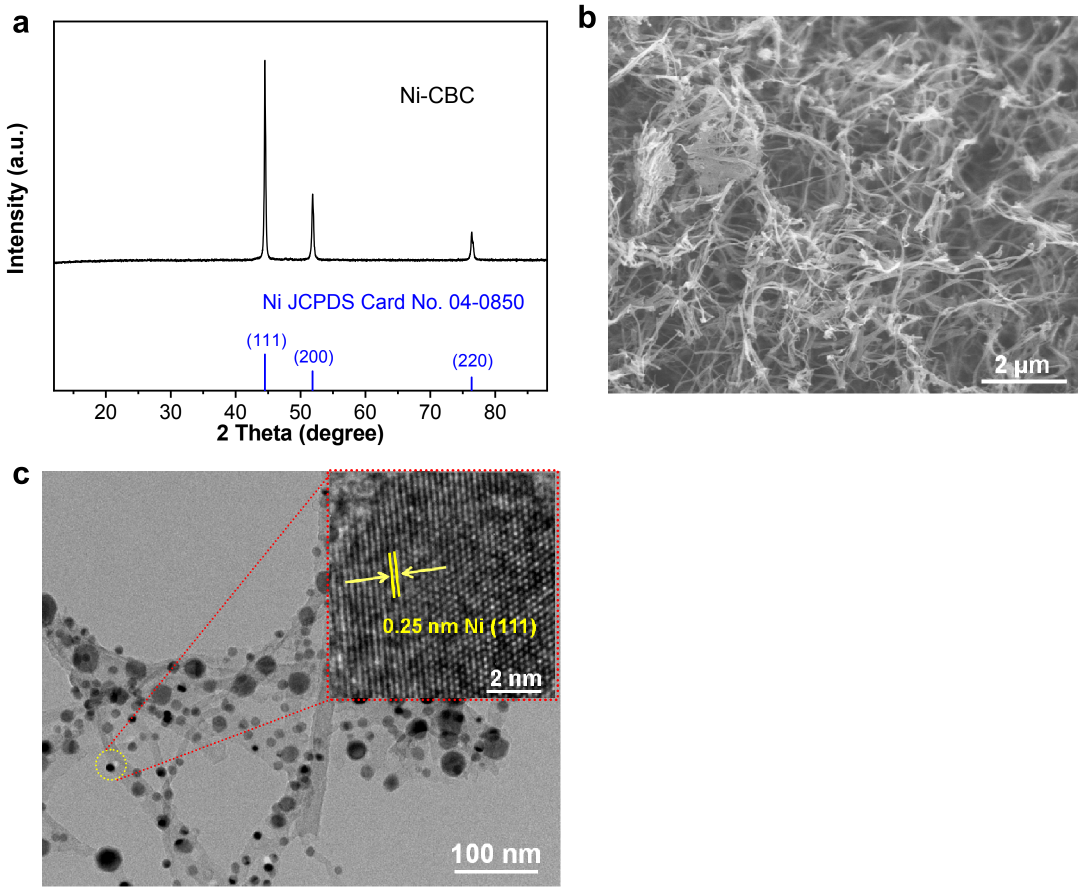

Figure S3. (**a**) XRD patterns, **(b)** SEM image and (**c**) TEM image (inset of HRTEM image) of Ni-CBC.

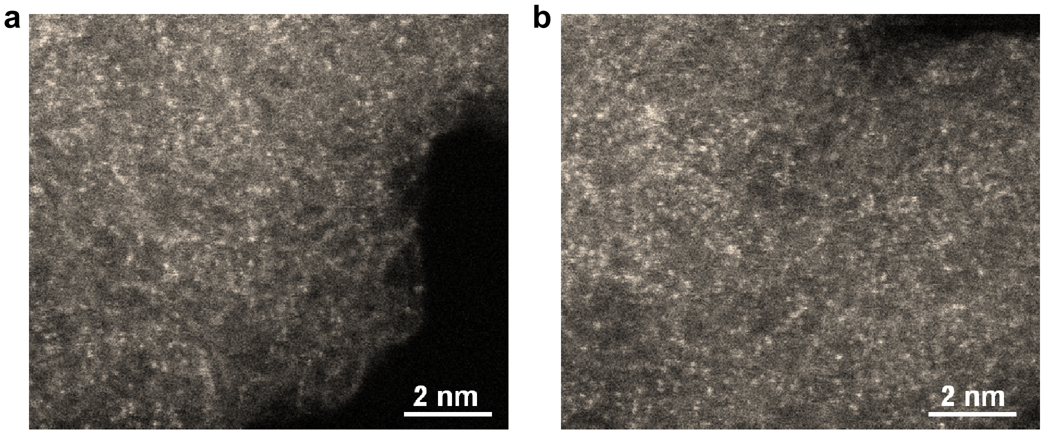

Figure S4. Aberration-corrected HAADF-STEM images obtained from different locations of Ni-O-C.

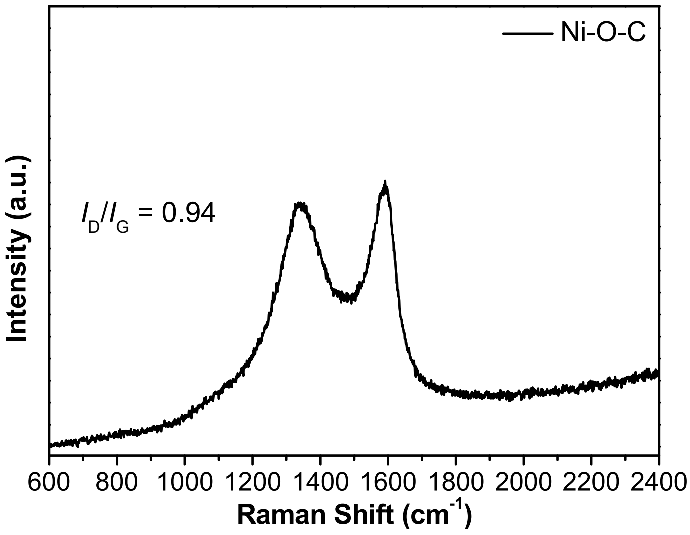

Figure S5. Raman spectrum of Ni-O-C.

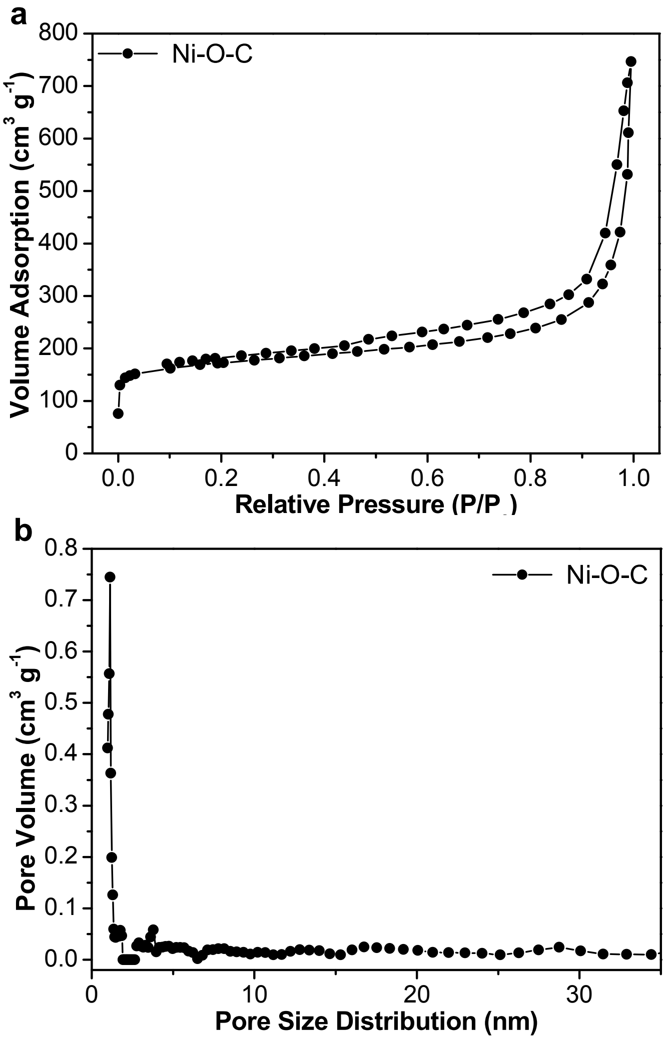

Figure S6. (**a**) N_2 adsorption-desorption isotherm and (**b**) Corresponding pore size distribution curve of Ni-O-C.

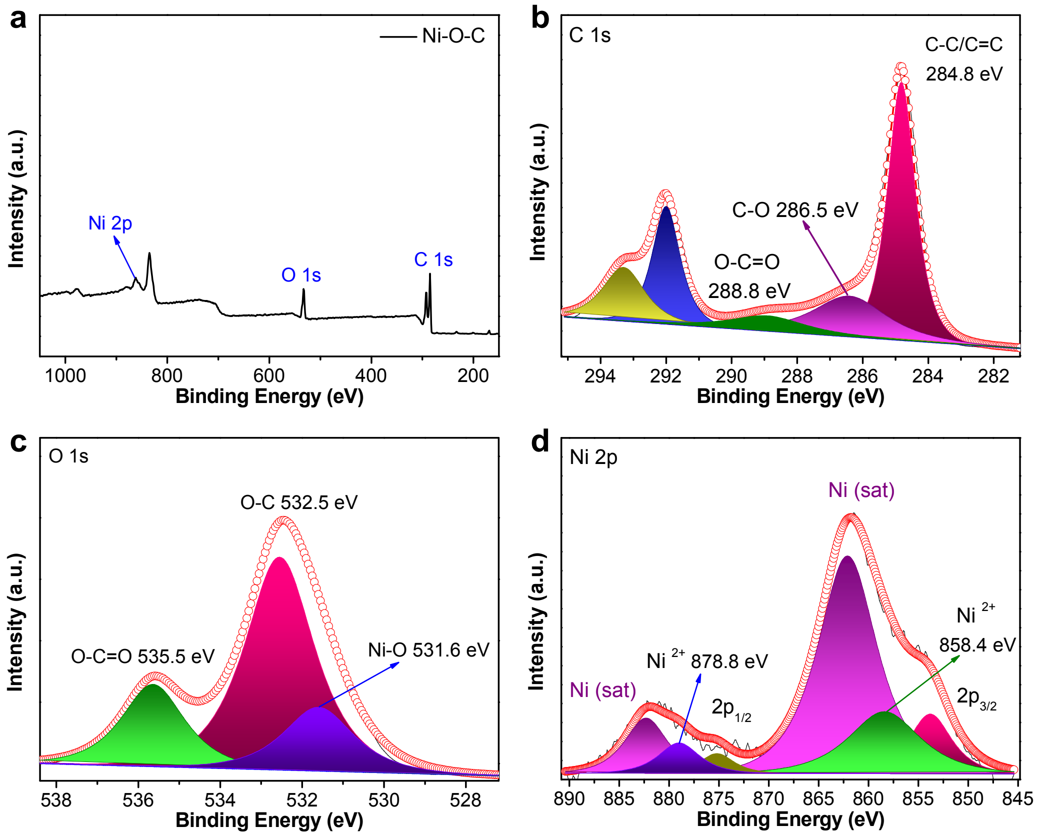

Figure S7. (**a**) Survey XPS spectrum and high-resolution XPS spectra of (**b**) C 1*s*, (**c**) O 1*s* and (**d**) Ni 2*p* of Ni-O-C.

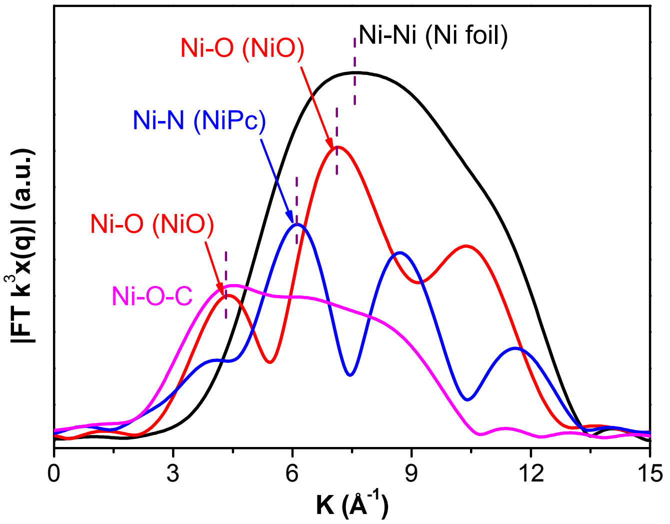

Figure S8. Comparison of q space magnitudes for k^3-weighted FT-EXAFS paths of different Ni-based samples.

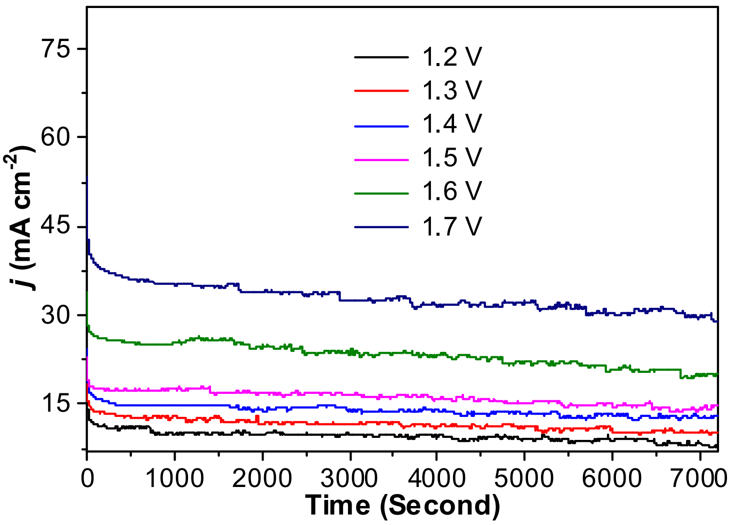

Figure S9. The chronoamperometric profiles of Ni-O-C at different potentials with 2 h of reaction for each measurement in 0.5 mmol benzene + 5.0 mmol H_2O_2 introduced Ar-saturated 0.1 M KOH electrolyte.

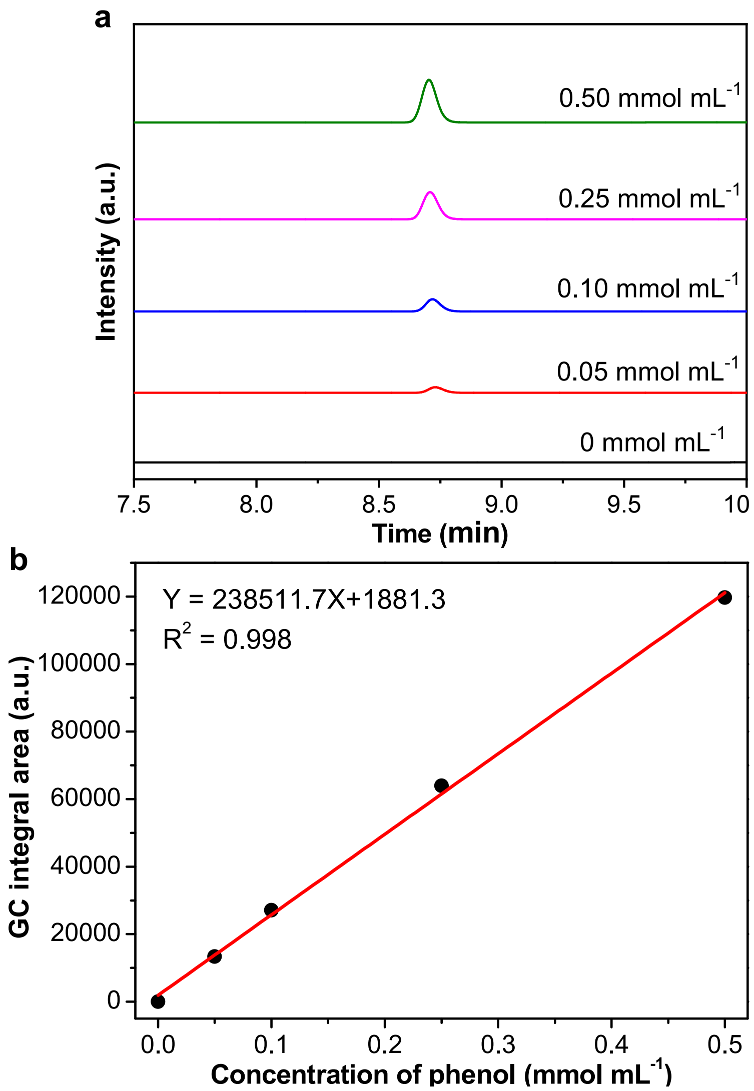

Figure S10. (**a**) The GC spectra obtained from the standard samples with different phenol concentrations of 0, 0.05, 0.10, 0.25 and 0.50 mmol mL^{-1}. (**b**) Calibration curve used to determine phenol concentrations.

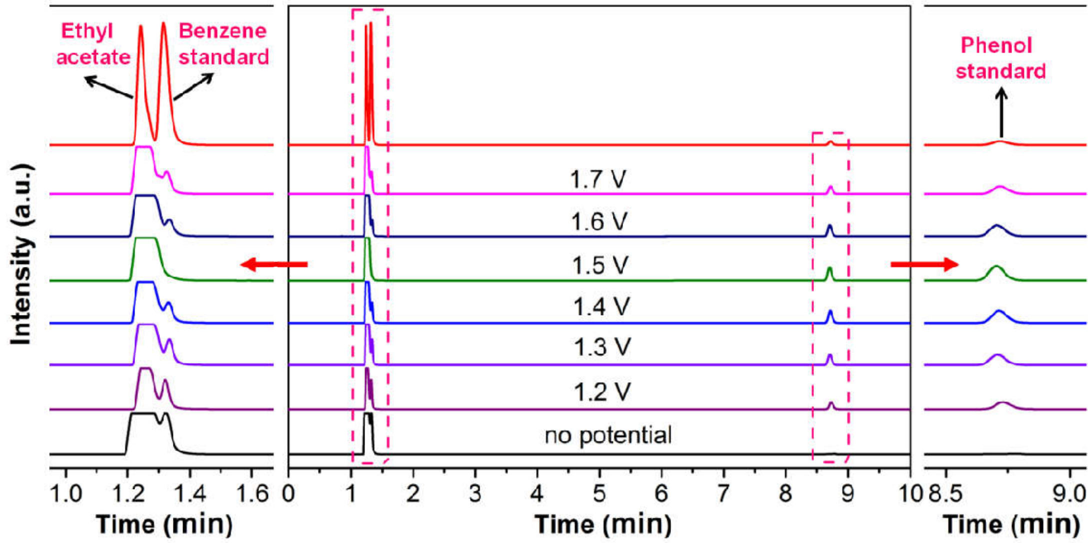

Figure S11. The GC spectra of the electrocatalytic samples obtained at different potentials with 2 h of reaction for each measurement in 0.5 mmol benzene + 5.0 mmol H_2O_2 introduced Ar-saturated 0.1 M KOH electrolyte. The concentration of benzene standard: 2.0 mmol mL^{-1}.

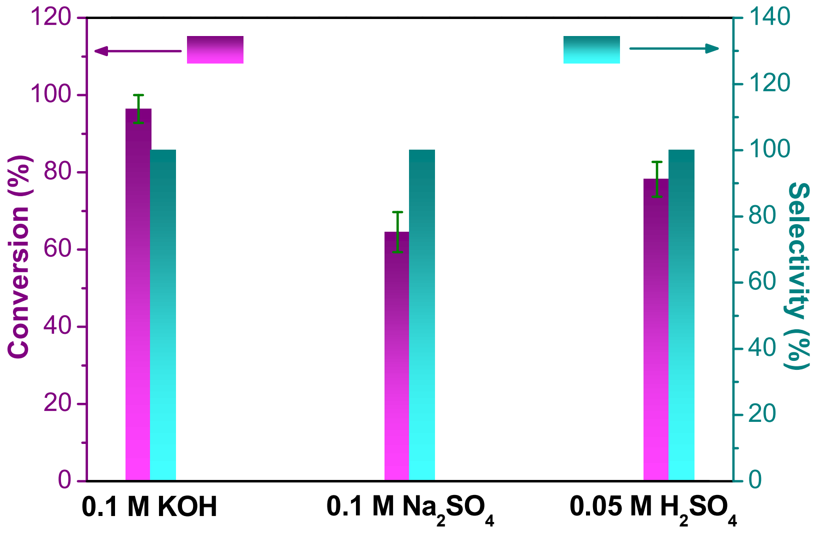

Figure S12. Effect of electrolyte pH on benzene oxidation catalyzed by Ni-O-C at 1.5 V (*vs.* RHE).

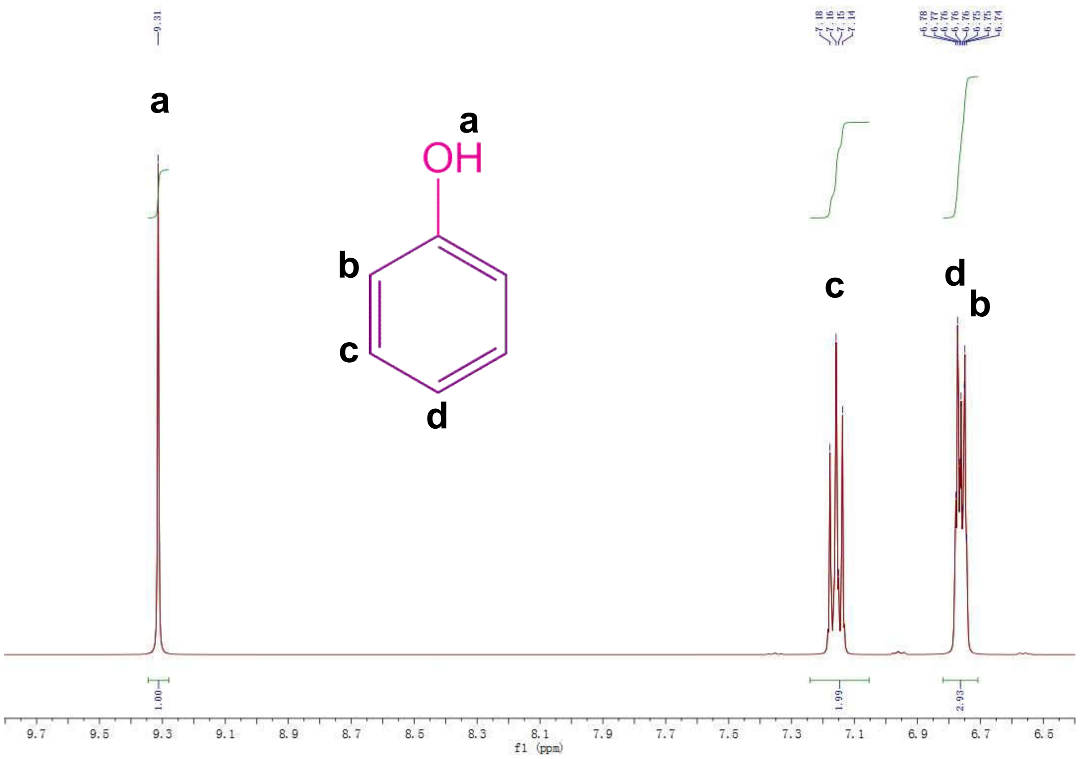

Figure S13. ^{1}H NMR spectrum of the product by the electrocatalytic oxidation of benzene at 1.5 V (*vs.* RHE). ^{1}H NMR (400 MHz, DMSO) δ [ppm]: 9.31 (s, 1 H), 7.16 (m, 2 H), 6.76 (m, 3 H).

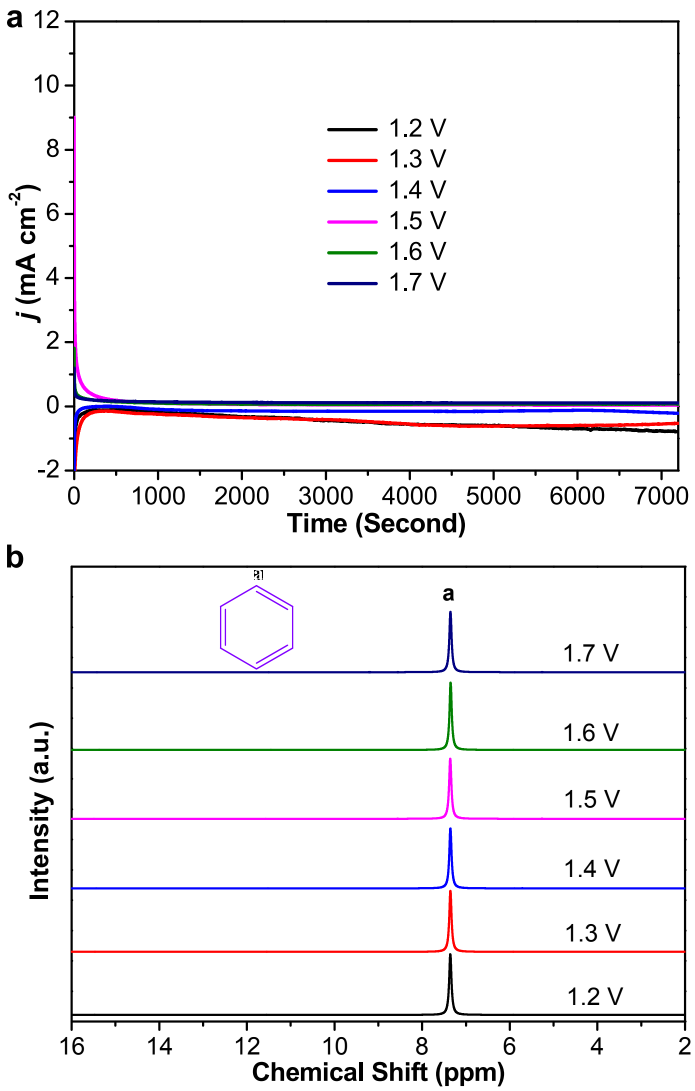

Figure S14. (**a**) The chronoamperometric profiles of Ni-O-C at different potentials with 2 h of reaction for each measurement in 0.5 mmol benzene introduced Ar-saturated 0.1 M KOH electrolyte. (**b**) The ^{1}H NMR spectra of the products corresponding to the applied potentials.

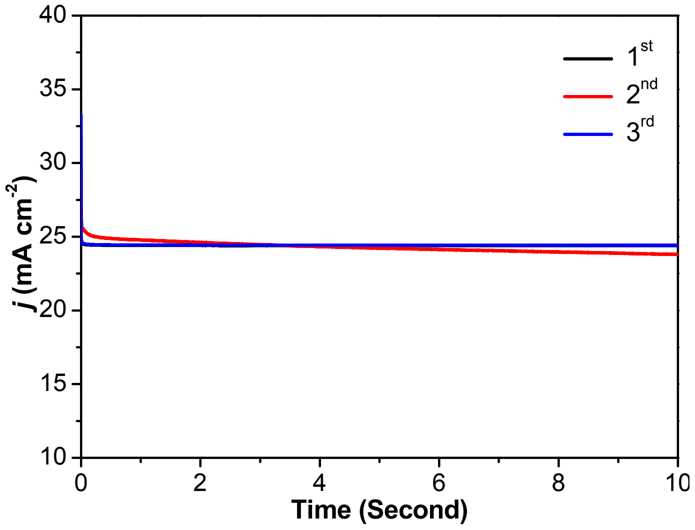

Figure S15. Long-time stability tests of Ni-O-C at 1.5 V (*vs.* RHE) for three replicated experiments.

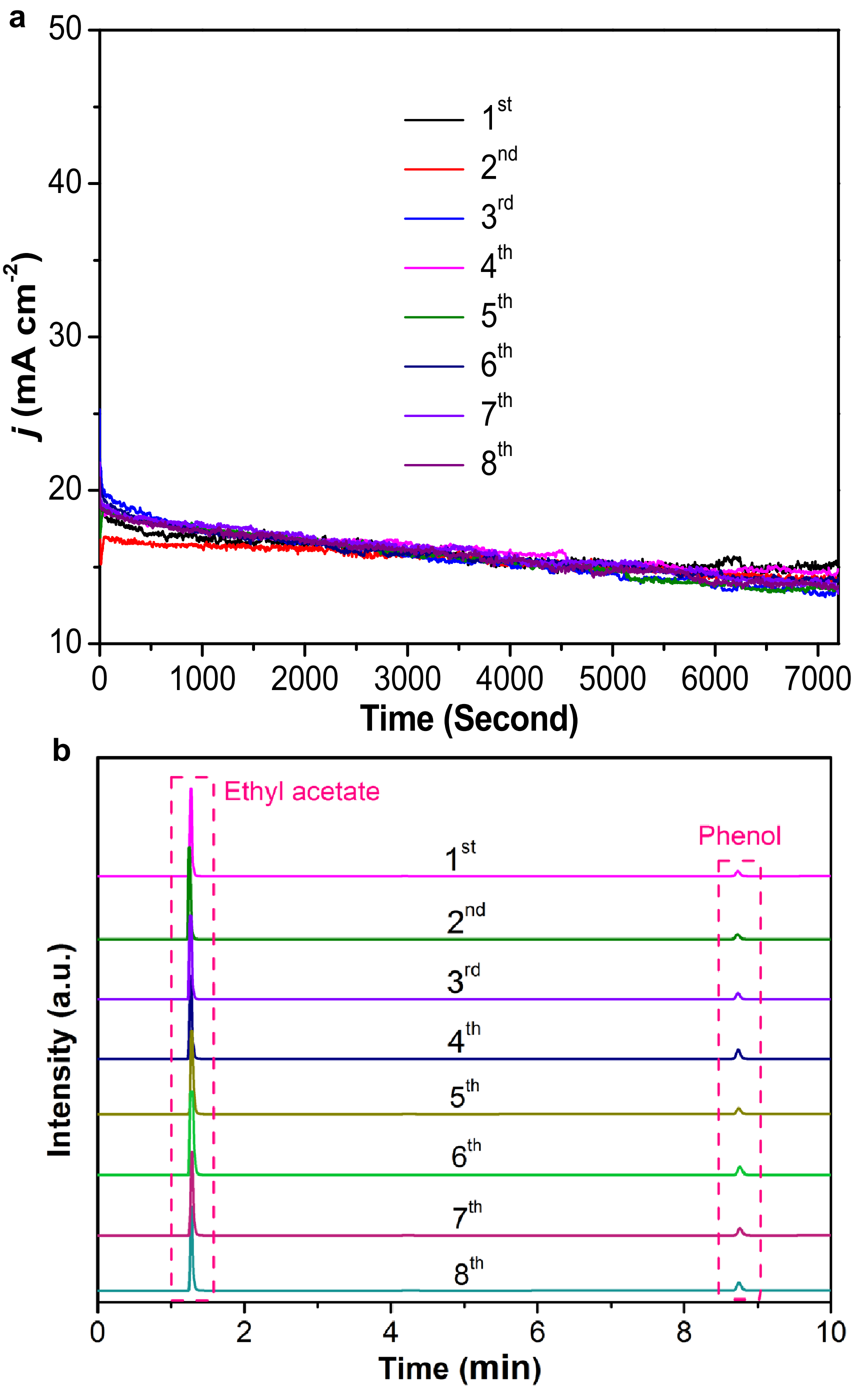

Figure S16. (**a**) Cycling stability test of Ni-O-C catalyzed benzene oxidation at 1.5 V (*vs.* RHE) for 8 consecutive cycles with 2 h period per cycle. (**b**) GC spectra of the electrocatalytic product after each testing cycle.

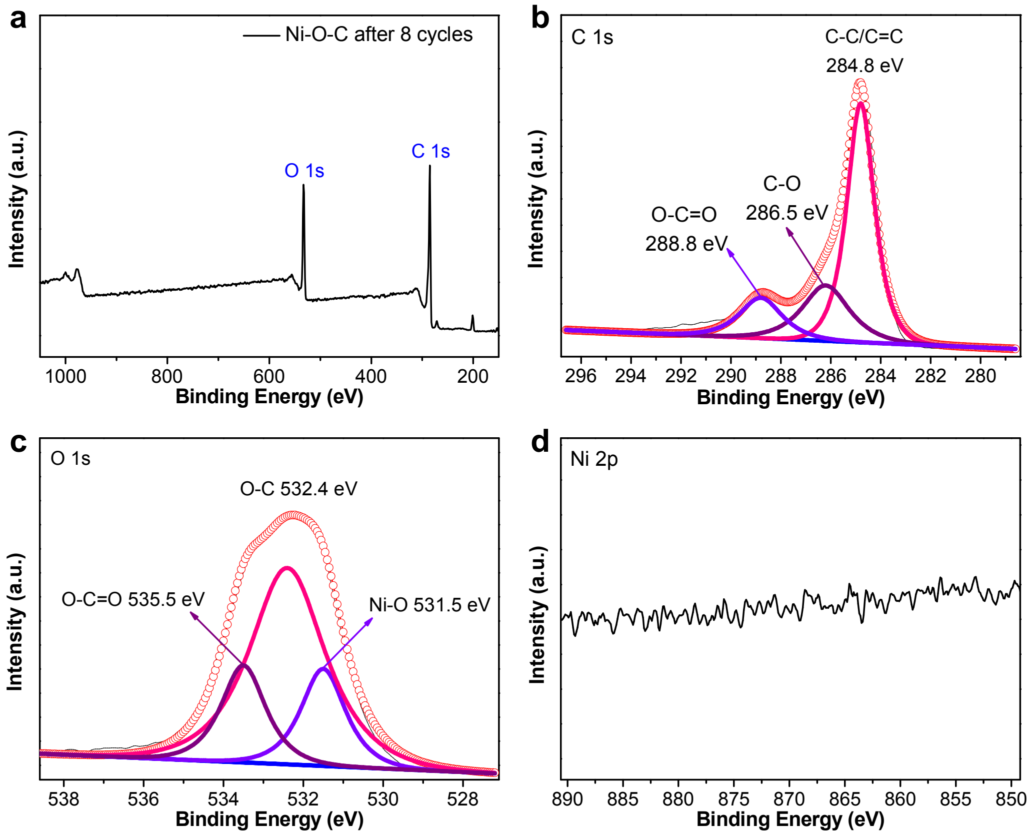

Figure S17. (**a**) Survey XPS spectrum and high-resolution XPS spectra of (**b**) C 1*s*, (**c**) O 1*s* and (**d**) Ni 2*p* of Ni-O-C after 8 cycles.

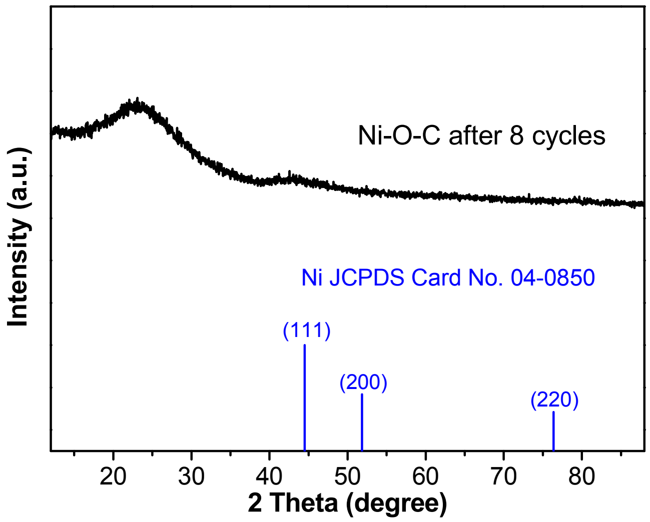

Figure S18. XRD patterns of Ni-O-C after 8 cycles.

After 8 cycles, the used Ni-O-C catalyst still exhibits that no metallic Ni or Ni-related oxides form. The XRD results unequivocally indicate that Ni-O-C catalyst possesses good structure stability.

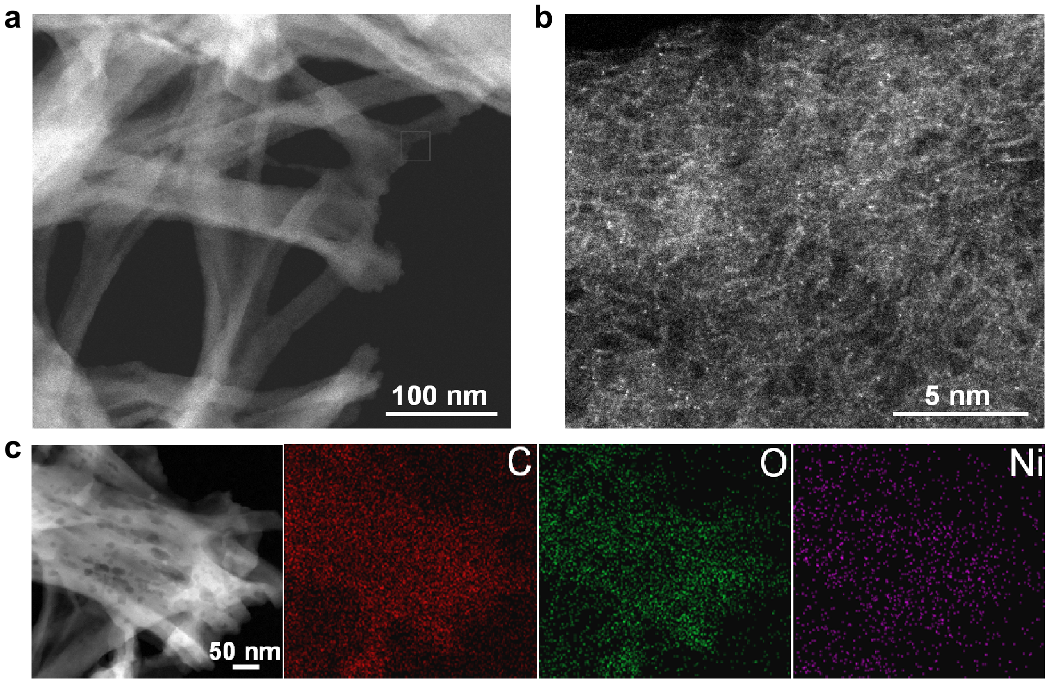

Figure S19. (**a, b**) Low- and high-magnification aberration-corrected HAADF-STEM images of Ni-O-C after 8 cycles. (**c**) The HAADF-STEM image and corresponding elemental mapping images of Ni-O-C after 8 cycles.

After 8 cycles, the used Ni-O-C catalyst still exhibits atomically dispersed nature of Ni, indicating high structural stability of the O-coordinated Ni active sites in Ni-O-C.

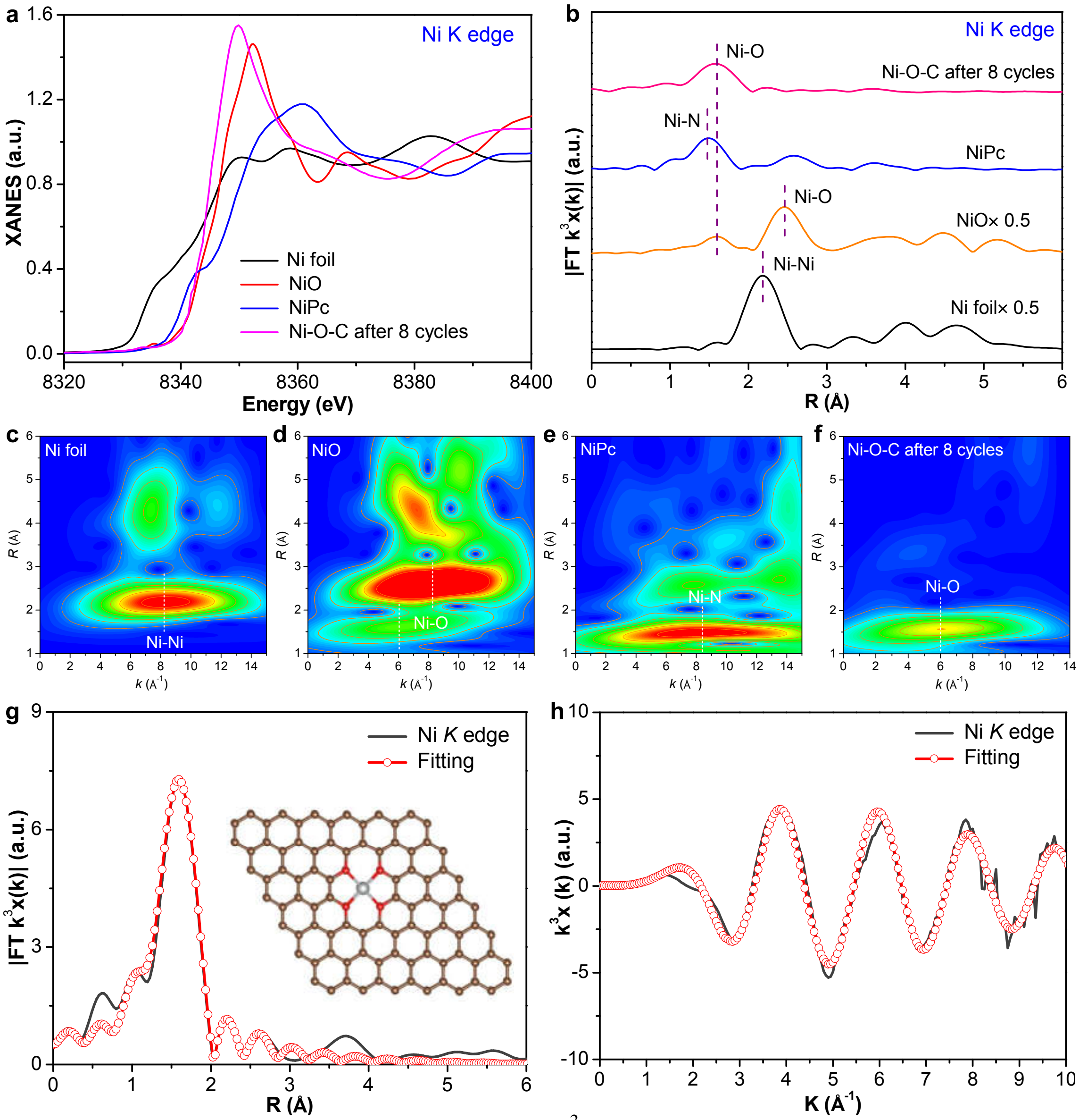

Figure S20. (**a**) Ni K edge XANES spectra and (**b**) k^3-weighted FT-EXAFS spectra of Ni-O-C after 8 cycles and references. (**c**)-(**f**) Ni K edge WT-EXAFS of Ni-O-C after 8 cycles and references. (**g**) and (**h**) Ni K edge EXAFS fitting curves of Ni-O-C after 8 cycles at R space and k space. Inset in (**g**) is the proposed Ni-(O-C_2)$_4$ coordination configuration in Ni-O-C after 8 cycles (Brown: C, red: O, White: Ni).

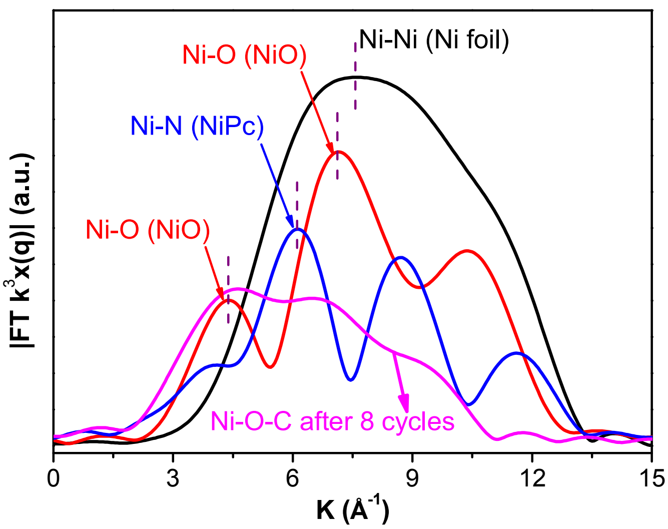

Figure S21. Comparison of *q* space magnitudes for k^3-weighted FT-EXAFS paths of Ni-O-C after 8 cycles and other Ni-based references.

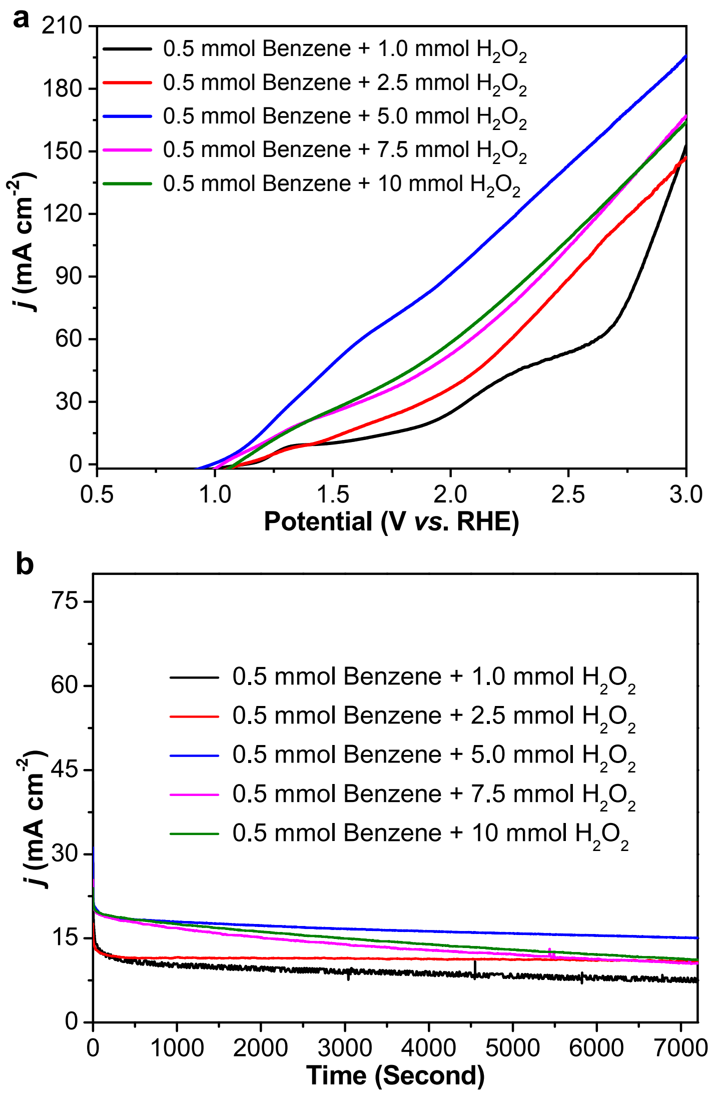

Figure S22. (**a**) LSV curves of Ni-O-C anode in 0.1 M KOH + 0.5 mmol benzene aqueous electrolyte containing different amounts of H_2O_2. (**b**) Time-dependent current density curves of Ni-O-C catalyzed benzene oxidation with different amounts of H_2O_2 at 1.5 V (*vs.* RHE).

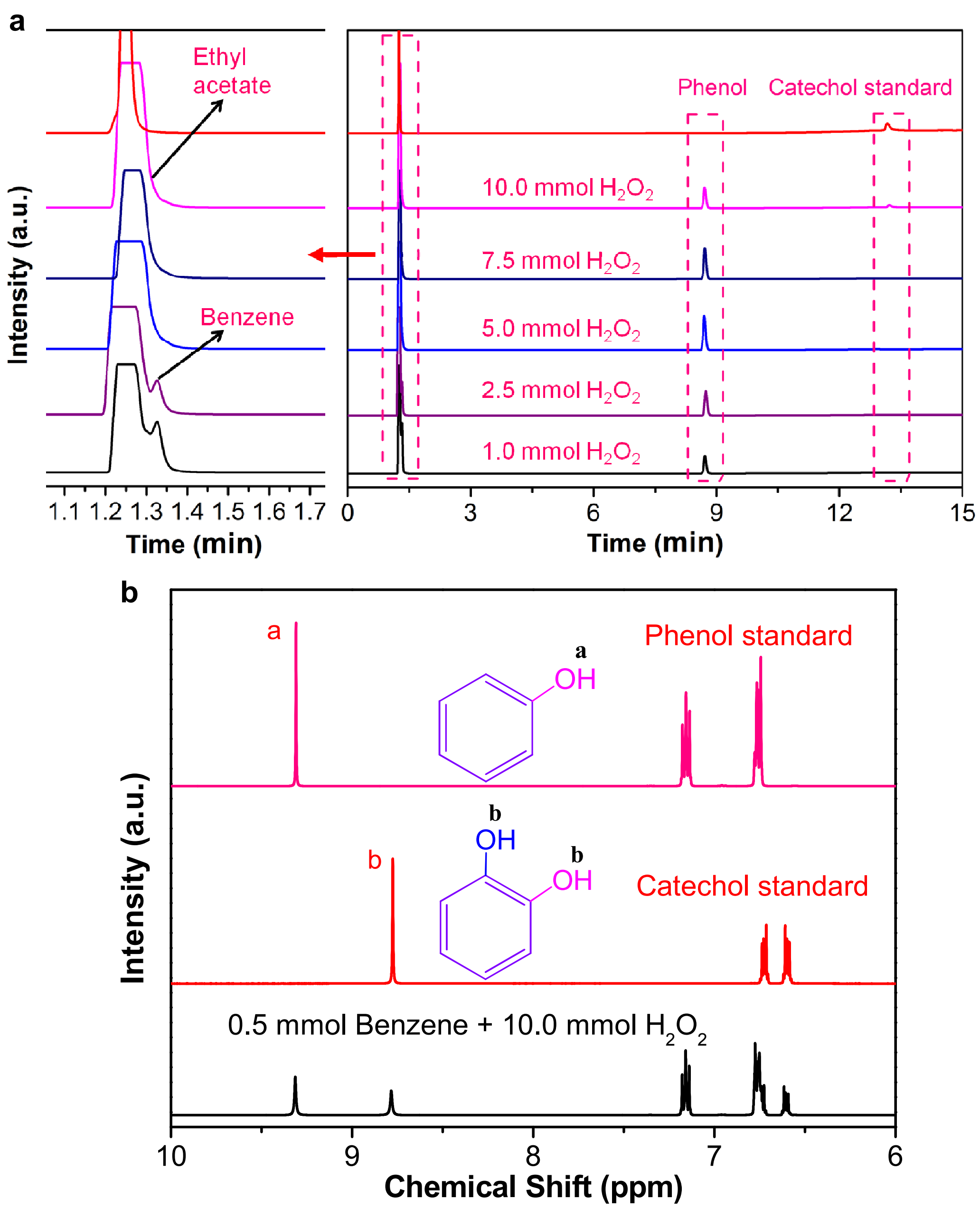

Figure S23. (**a**) GC spectra of the products corresponding to different amount of H_2O_2 (1.0, 2.0, 5.0, 7.5 and 10.0 mmol) in benzene (0.5 mmol) oxidation catalyzed by Ni-O-C at 1.5 V (*vs.* RHE). (**b**) The ^{1}H NMR spectra of the product corresponding to 0.5 mmol benzene and 10.0 mmol H_2O_2 catalyzed by Ni-O-C at 1.5 V (*vs.* RHE).

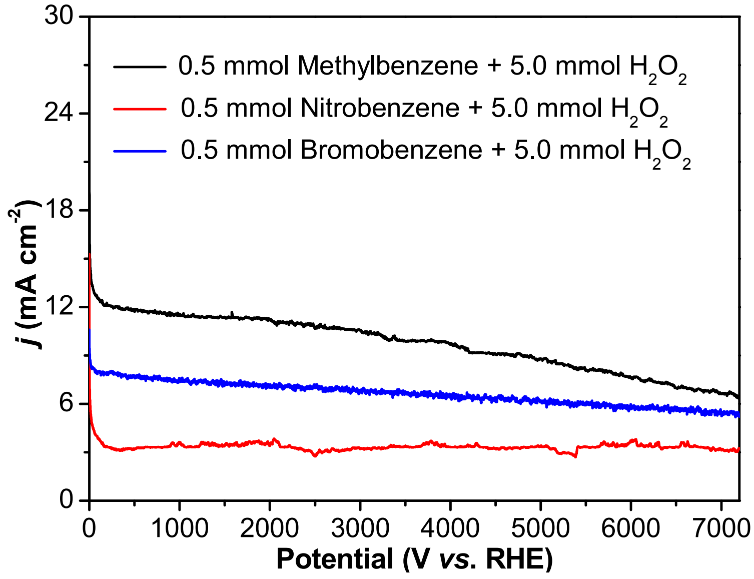

Figure S24. Time-dependent current density curves of Ni-O-C catalyzed aromatic compound oxidation including methylbenzene, nitrobenzene and bromobenzene at 1.5 V (*vs.* RHE).

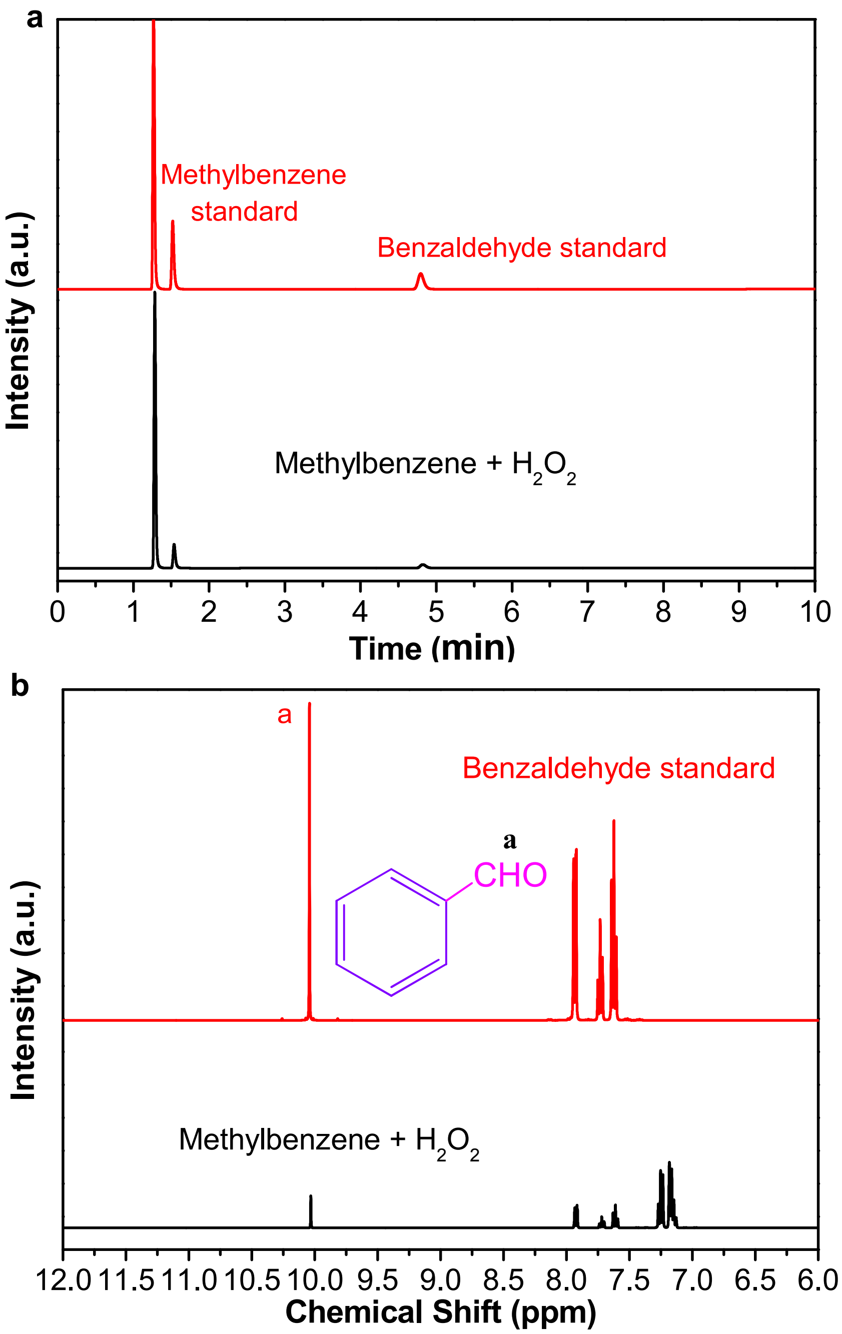

Figure S25. (**a**) GC spectra of methylbenzene, benzaldehyde standards the product corresponding to methylbenzene oxidation catalyzed by Ni-O-C at 1.5 V (*vs.* RHE). (**b**) The ^{1}H NMR spectra of methylbenzene, benzaldehyde standards and the product corresponding to methylbenzene oxidation catalyzed by Ni-O-C at 1.5 V (*vs.* RHE).

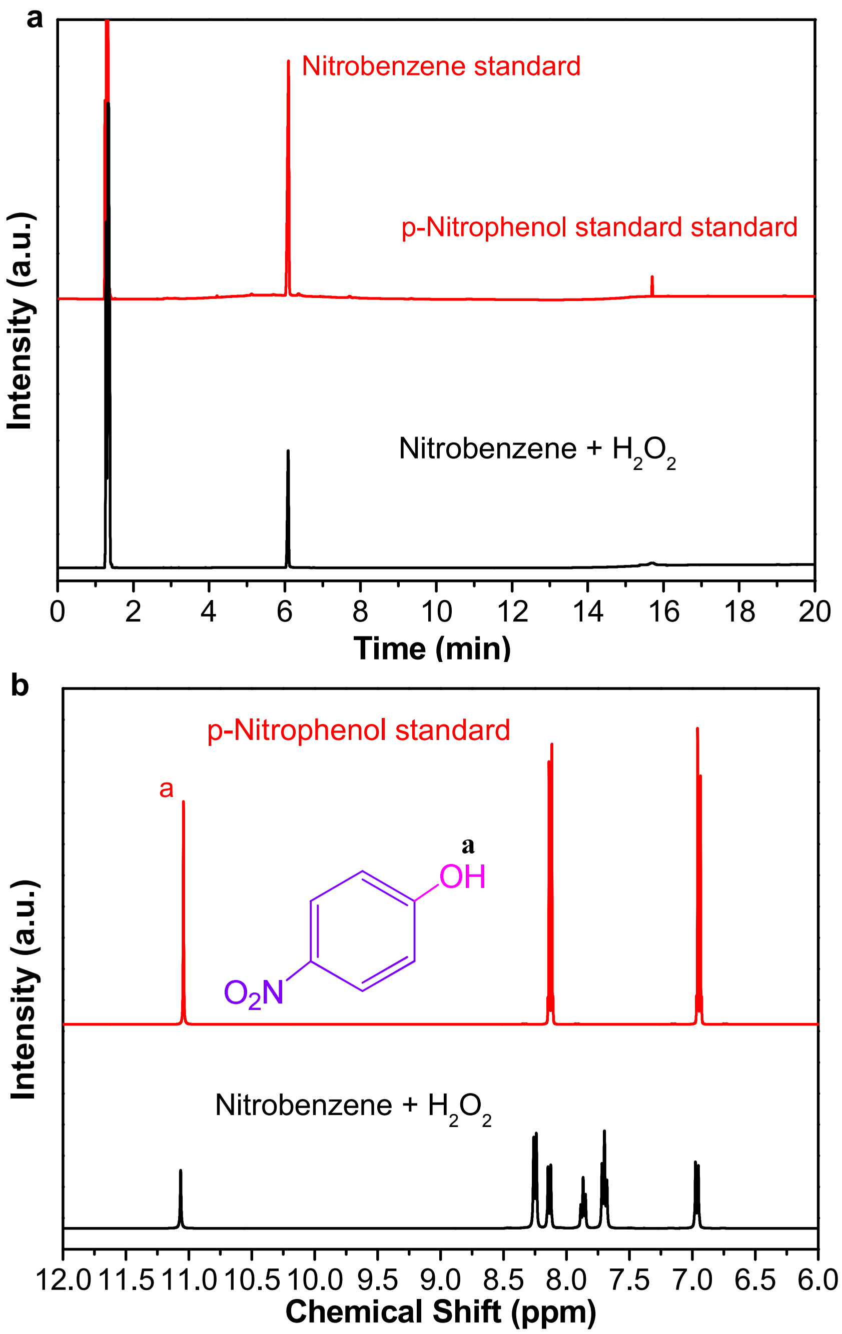

Figure S26. (**a**) GC spectra of nitrobenzene, p-nitrophenol standards and the product corresponding to nitrobenzene oxidation catalyzed by Ni-O-C at 1.5 V (*vs.* RHE). (**b**) The ^{1}H NMR spectra of nitrobenzene, p-nitrophenol standards and the product corresponding to nitrobenzene oxidation catalyzed by Ni-O-C at 1.5 V (*vs.* RHE).

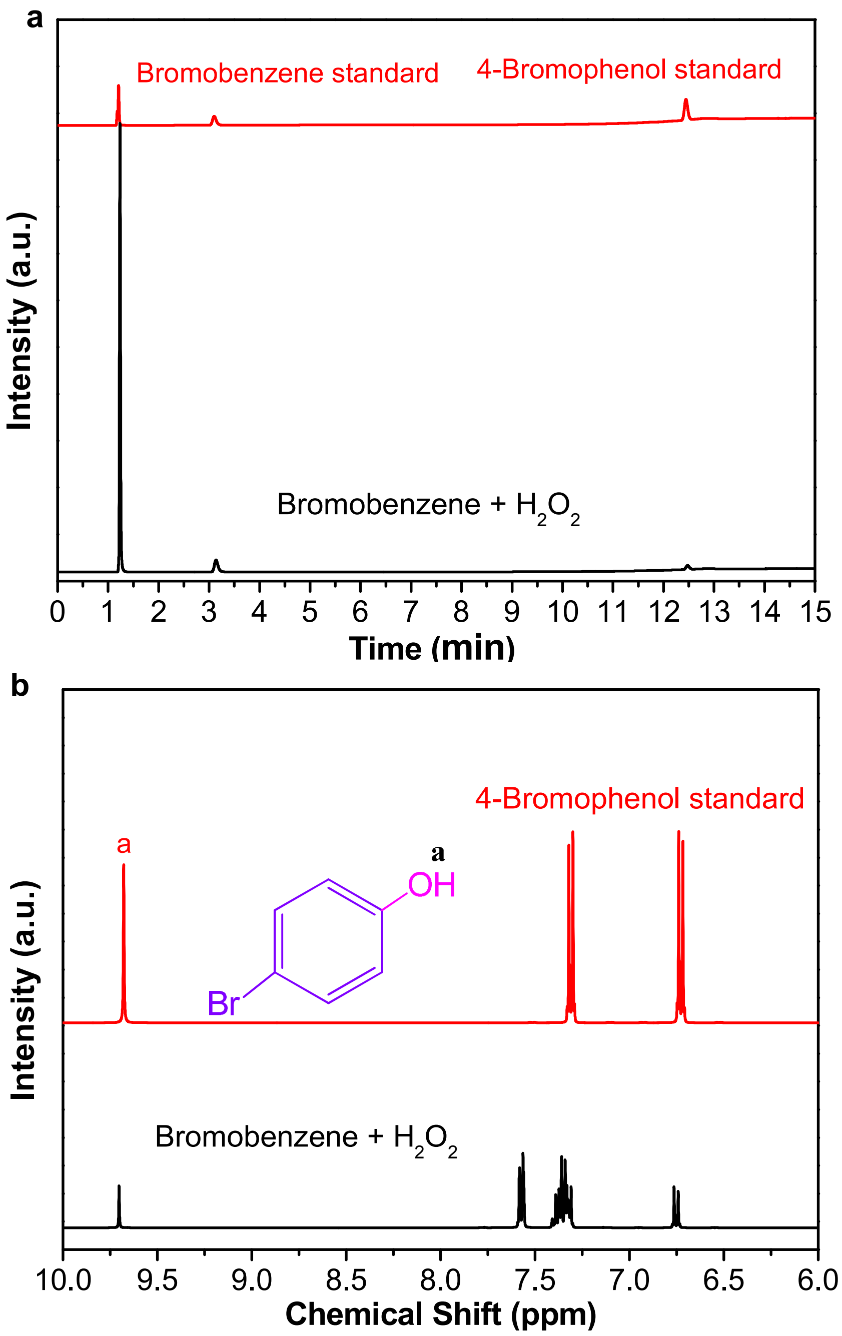

Figure S27. (**a**) GC spectra of bromobenzene, 4-bromophenol standards and the product corresponding to bromobenzene oxidation catalyzed by Ni-O-C at 1.5 V (*vs.* RHE). (**b**) The ^{1}H NMR spectra of bromobenzene, 4-bromophenol standards and the product corresponding to bromobenzene oxidation catalyzed by Ni-O-C at 1.5 V (*vs.* RHE).

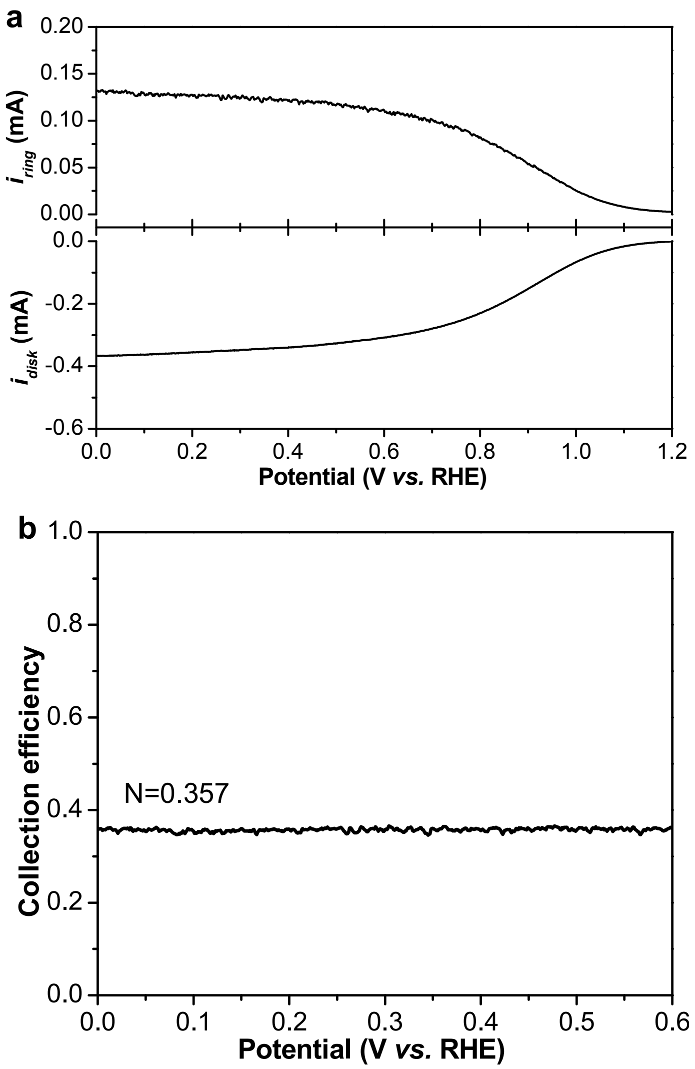

Figure S28. (**a**) LSV curves of the bare RRDE in Ar-saturated 0.1 M KOH aqueous electrolyte containing 2.0 mmol $K_3[Fe(CN)_6]$ at 1600 rpm to calibrate the collection efficiency. (**b**) Calculated collection efficiency (N) based on LSV result *via* dividing the ring current by the disk current.

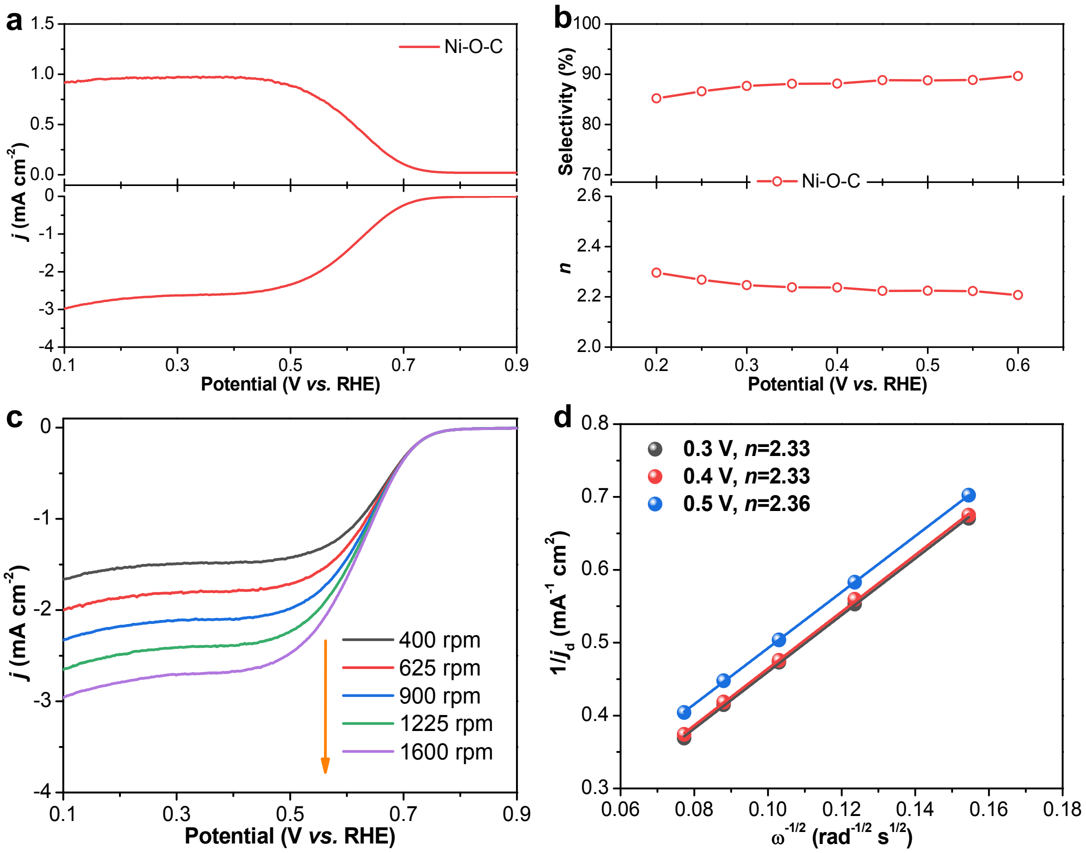

Figure S29. (**a**) LSV curve of Ni-O-C at 1600 rpm in O_2-saturated 0.1 M KOH electrolyte. (**b**) Calculated H_2O_2 selectivity and electron transfer number (n) based on RRDE measurements. (**c**) LSV cures of Ni-O-C at different rotation speeds in 0.1 M KOH electrolyte. (**d**) The Koutecky-Levich (*K-L*) plots and corresponding n value of Ni-O-C.

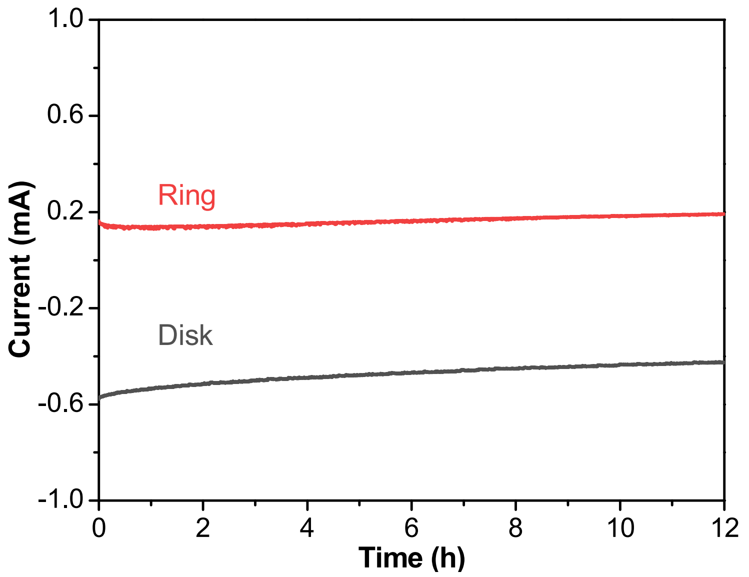

Figure S30. Stability measurement of Ni-O-C at a fixed disk potential of 0.4 V (*vs.* RHE).

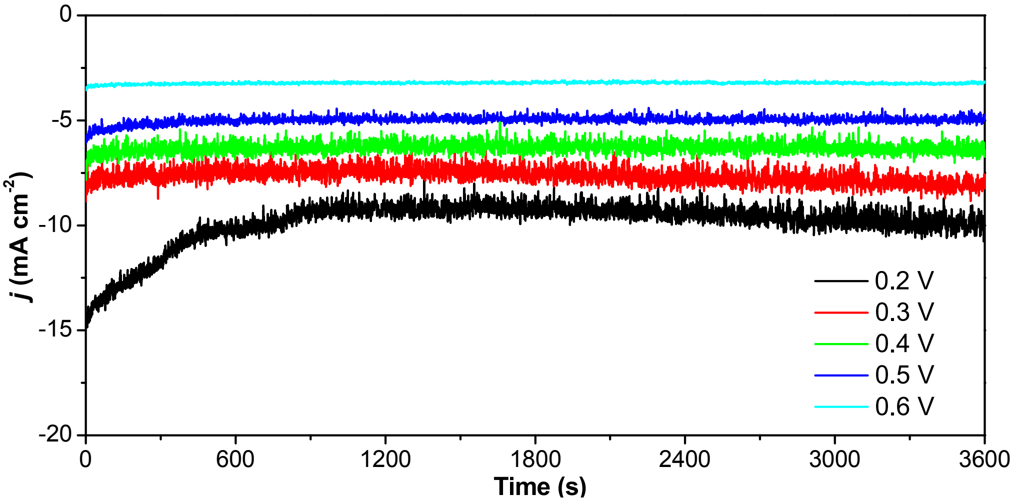

Figure S31. Time-dependent current density curves of Ni-O-C catalyzed ORR at different potentials.

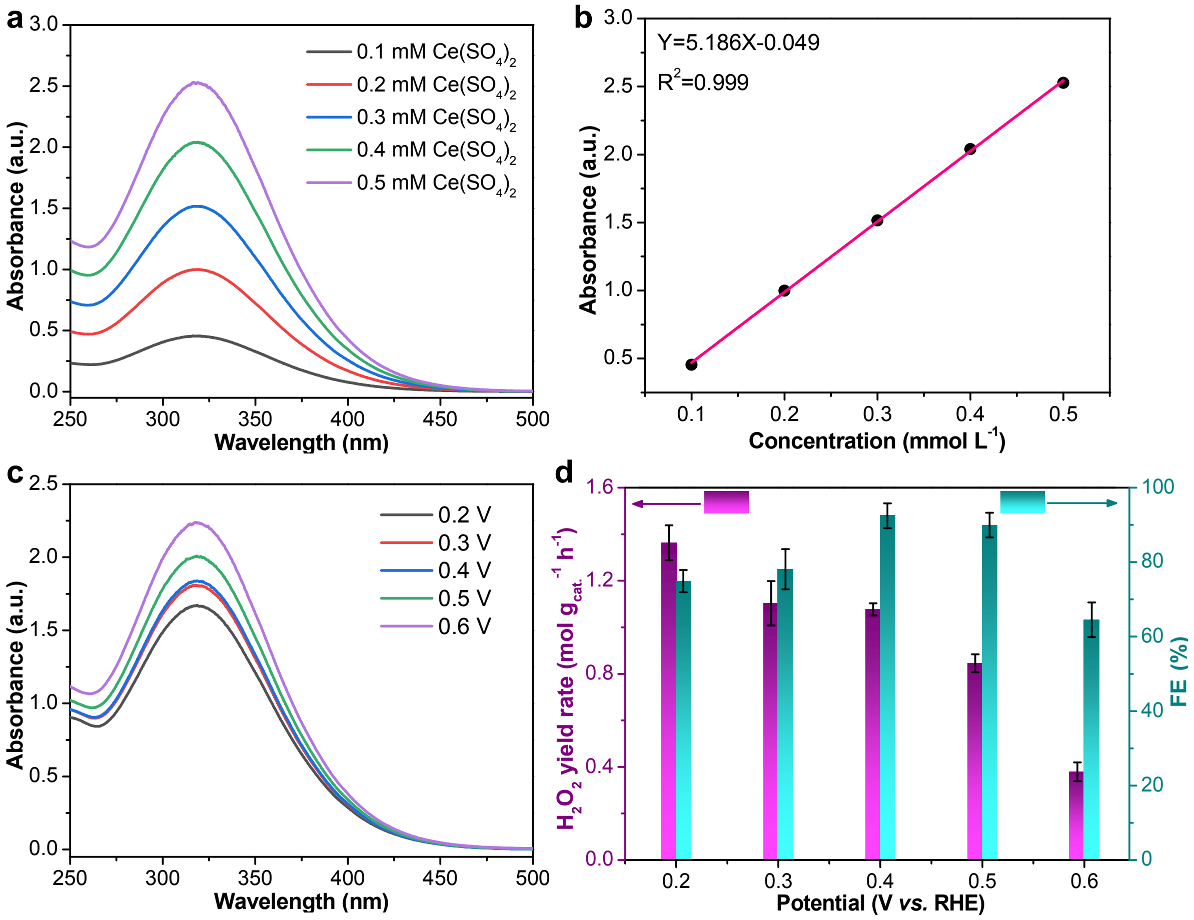

Figure S32. (**a**) UV-vis spectra of Ce^{4+} solution with various concentrations and (**b**) The corresponding standard curve. (**c**) UV-vis absorption spectra of Ce^{4+} solutions after injecting electrolytes at different potentials. (**d**) Dependence of H_2O_2 yield rate and FE on the applied potentials.

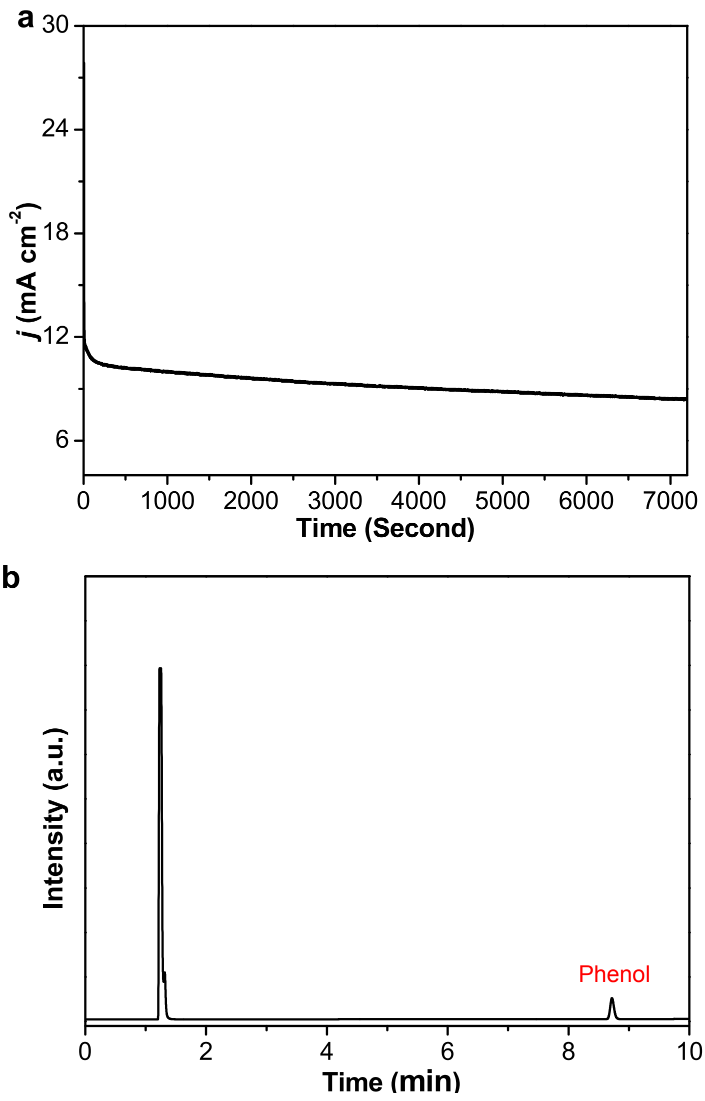

Figure S33. (**a**) Time-dependent current density curve of Ni-O-C catalyzed 0.5 mmol benzene oxidation with 1.1 mmol H_2O_2 at 1.5 V (*vs*. RHE). (**b**) GC spectra of the product corresponding to Ni-O-C catalyzed 0.5 mmol benzene oxidation with 1.1 mmol H_2O_2 at 1.5 V (*vs*. RHE).

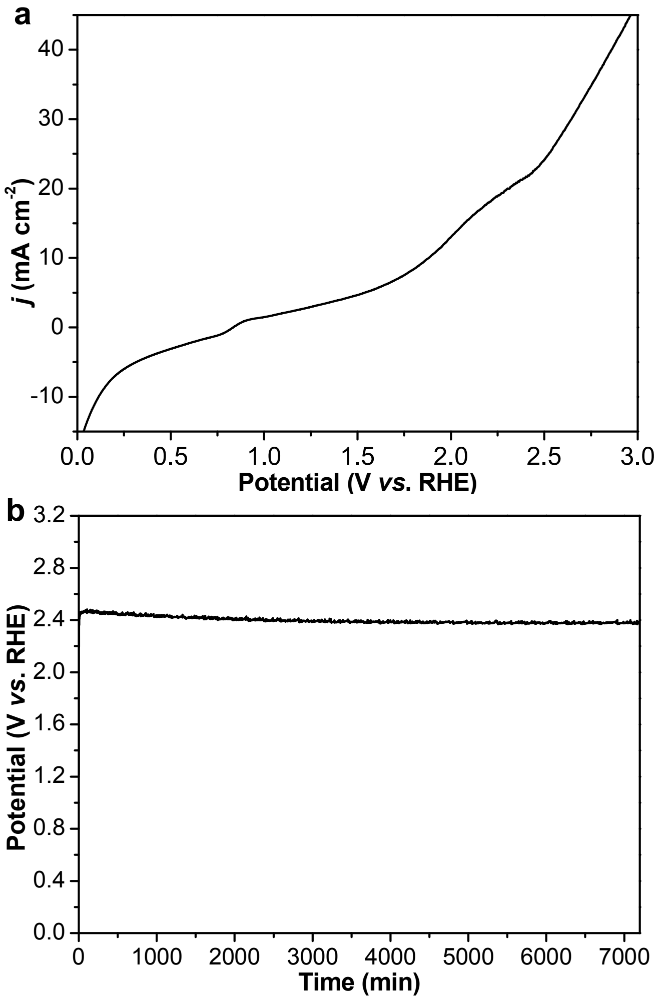

Figure S34. (**a**) LSV curve of Ni-O-C anode in O_2-saturated 0.1 M KOH with 0.5 mmol benzene. (**b**) The chronopotentiometry profile of Ni-O-C at a current density of 10 mA cm^{-2} in O_2-saturated 0.1 M KOH electrolyte with 0.5 mmol benzene.

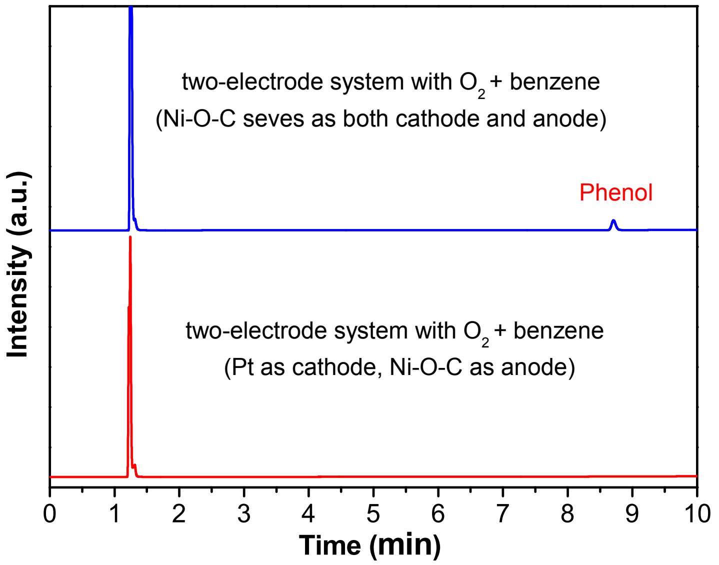

Figure S35. GC spectra of electrocatalytic products under different reaction conditions in a single cell at a current density of 10 mA cm^{-2}.

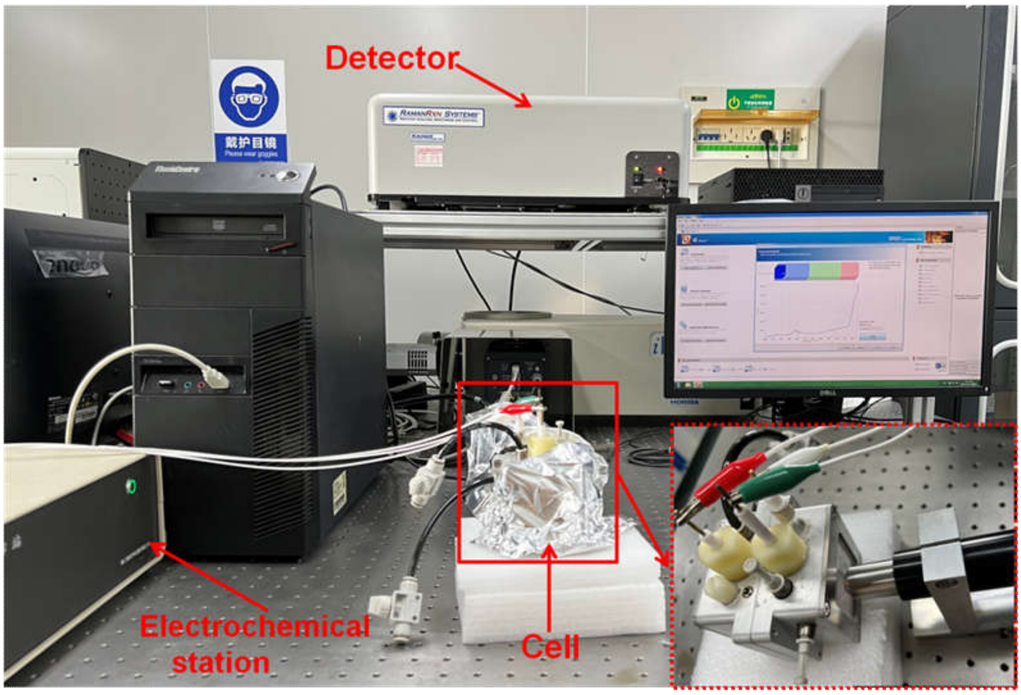

Figure S36. The experimental setup for the *Operando* Raman measurements.

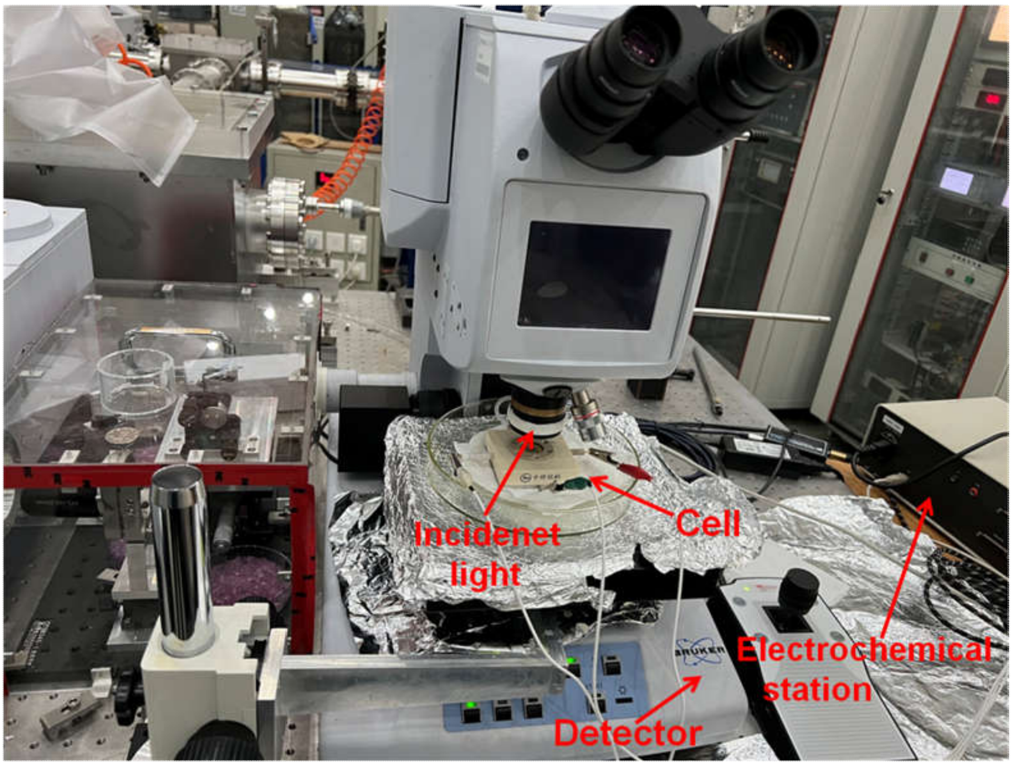

Figure S37. The experimental setup for the *Operando* synchrotron radiation FTIR measurements.

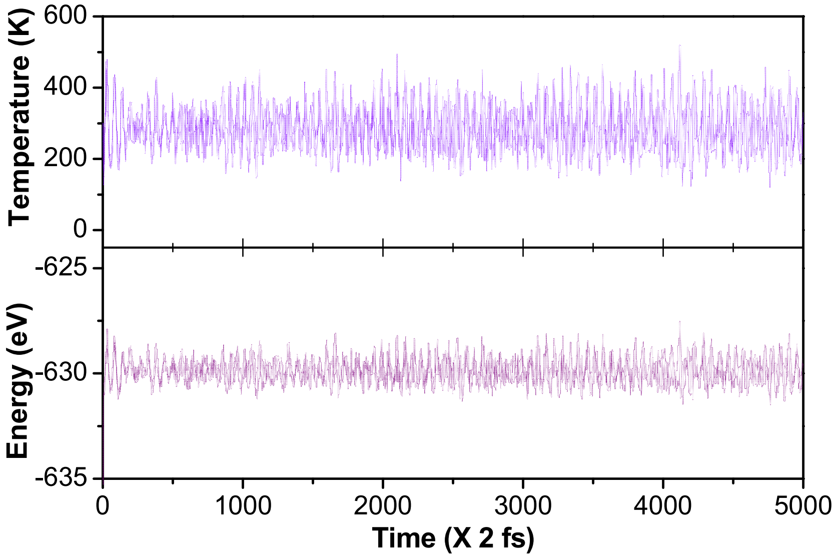

Figure S38. Variations of temperature and energy *vs* time for AIMD simulations of Ni-(O-C_2)$_4$. The simulation is run under 300 K for 10 ps with a time step of 2 fs.

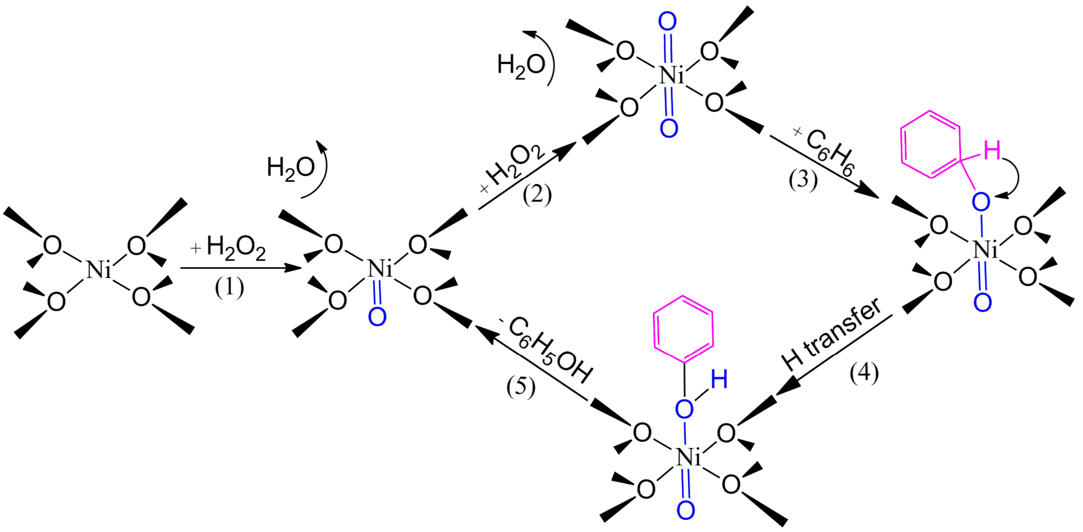

Figure S39. Scheme for the reaction mechanism of the electrocatalytic oxidation of benzene to phenol on Ni-(O-C_2)$_4$.

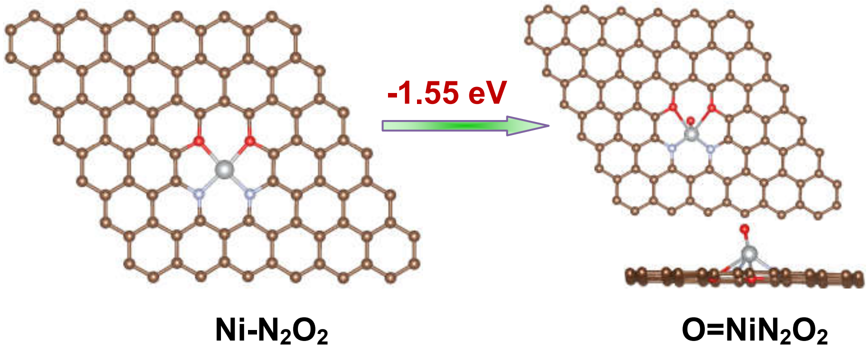

Figure S40. DFT calculated intermediates structures on Ni-N_2O_2 (Brown: C, red: O, White: Ni).

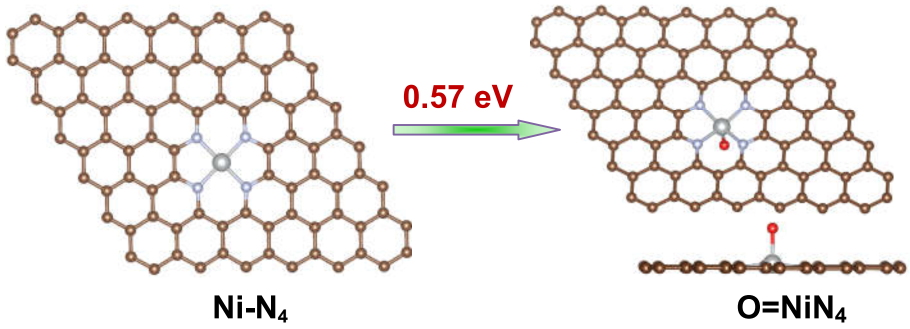

Figure S41. DFT calculated intermediates structures on Ni-N_4 (Brown: C, red: O, White: Ni).

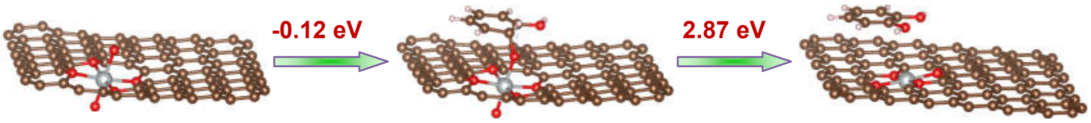

Figure S42. DFT calculated intermediates structures of the foramtion of catechol on Ni-(O-C_2)$_4$ (Brown: C, red: O, White: Ni, Silver: H).

References

[1] B. Lee, H. Naito, T. Hibino, *Angew. Chem. Int. Ed.* **2012**, 51, 440.

[2] H. W. Kim, M. B. Ross, N. Kornienko, L. Zhang, J. Guo, P. Yang, B. D. McCloskey, *Nat. Catal.* **2018**, 1, 282.

[3] G. Kresse, J. Furthmüller, *Phys. Rev. B* **1996**, 54, 11169.

[4] J. P. Perdew, J. A. Chevary, S. H. Vosko, K. A. Jackson, M. R. Pederson, D. J. Singh, C. Fiolhais, *Phys. Rev. B* **1992**, 46, 6671.

[5] J. K. Nørskov, J. Rossmeisl, A. Logadottir, L. Lindqvist, J. R. Kitchin, T. Bligaard, H. Jonsson, *J. Phys. Chem. B* **2004**, 108, 17886.

[6] J. Moellmann, S. Grimme, *J. Phys. Chem. C* **2014**, 118, 7615.

[7] M. M. Baker, G. Jenkins, *Adv. Catal.* **1955**, 7, 1.

[8] V. Wang, N. Xu, J.-C. Liu, G. Tang, W.-T. Geng, *Comput. Phys. Commun.* **2021**, 267, 108033.

[9] E. Jung, H. Shin, B.-H. Lee, V. Efremov, S. Lee, H. S. Lee, J. Kim, W. Hooch Antink, S. Park, K.-S. Lee, S.-P. Cho, J. S. Yoo, Y.-E. Sung, T. Hyeon, *Nat. Mater.* **2020**, 19, 436.

[10] B. Q. Li, C. X. Zhao, J. N. Liu, Q. Zhang, *Adv. Mater.* **2019**, 31, 1808173.

[11] W. Liu, C. Zhang, J. Zhang, X. Huang, M. Song, J. Li, F. He, H. Yang, J. Zhang, D. Wang, *Appl. Catal. B: Environ.* **2022**, 310, 121312.

[12] B.-W. Zhang, T. Zheng, Y.-X. Wang, Y. Du, S.-Q. Chu, Z. Xia, R. Amal, S.-X. Dou, L. Dai, *Commun. Chem.* **2022**, 5, 1.

[13] C. Tang, L. Chen, H. Li, L. Li, Y. Jiao, Y. Zheng, H. Xu, K. Davey, S.-Z. Qiao, *J. Am. Chem. Soc.* **2021**, 143, 7819.

[14] Y. Jia, Z. Xue, J. Yang, Q. Liu, J. Xian, Y. Zhong, Y. Sun, X. Zhang, Q. Liu, D. Yao, *Angew. Chem. Int. Ed.* **2022**, 61, e202110838.

[15] S. Yang, J. Kim, Y. J. Tak, A. Soon, H. Lee, *Angew. Chem. Int. Ed.* **2016**, 55, 2058.

[16] S. Yang, Y. J. Tak, J. Kim, A. Soon, H. Lee, *ACS Catal.* **2017**, 7, 1301.

[17] R. Shen, W. Chen, Q. Peng, S. Lu, L. Zheng, X. Cao, Y. Wang, W. Zhu, J. Zhang, Z. Zhuang, *Chem* **2019**, 5, 2099.

[18] Q. Yang, W. Xu, S. Gong, G. Zheng, Z. Tian, Y. Wen, L. Peng, L. Zhang, Z. Lu, L. Chen, *Nat. Commun.* **2020**, 11, 1.

[19] F. Zhang, Y. Zhu, C. Tang, Y. Chen, B. Qian, Z. Hu, Y. C. Chang, C. W. Pao, Q. Lin, S. A. Kazemi, *Adv. Funct. Mater.* **2021**, 2110224.

[20] C. Tang, Y. Jiao, B. Shi, J. N. Liu, Z. Xie, X. Chen, Q. Zhang, S. Z. Qiao, *Angew.*

Chem. Int. Ed. **2020**, 59, 9171.

[21] H. Sheng, A. N. Janes, R. D. Ross, D. Kaiman, J. Huang, B. Song, J. Schmidt, S. Jin, *Energy Environ. Sci.* **2020**, 13, 4189.

[22] H. Sheng, E. D. Hermes, X. Yang, D. Ying, A. N. Janes, W. Li, J. Schmidt, S. Jin, *ACS Catal.* **2019**, 9, 8433.

[23] L. Zhang, J. Liang, L. Yue, Z. Xu, K. Dong, Q. Liu, Y. Luo, T. Li, X. Cheng, G. Cui, *Nano Research* **2022**, 15, 304.

[24] X. L. Zhang, X. Su, Y. R. Zheng, S. J. Hu, L. Shi, F. Y. Gao, P. P. Yang, Z. Z. Niu, Z. Z. Wu, S. Qin, *Angew. Chem. Int. Ed.* **2021**, 60, 26922.

[25] F. Xia, B. Li, Y. Liu, Y. Liu, S. Gao, K. Lu, J. Kaelin, R. Wang, T. J. Marks, Y. Cheng, *Adv. Funct. Mater.* **2021**, 31, 2104716.

[26] M. Wang, X. Dong, Z. Meng, Z. Hu, Y. G. Lin, C. K. Peng, H. Wang, C. W. Pao, S. Ding, Y. Li, *Angew. Chem. Int. Ed.* **2021**, 60, 11190.

[27] A. Byeon, J. Cho, J. M. Kim, K. H. Chae, H.-Y. Park, S. W. Hong, H. C. Ham, S. W. Lee, K. R. Yoon, J. Y. Kim, *Nanoscale Horiz.* **2020**, 5, 832.

[28] Y. Chang, J. Li, J. Ma, Y. Liu, R. Xing, Y. Wang, G. Zhang, *Sci. China Mater.* **2022**, 1.

[29] S. Chen, T. Luo, K. Chen, Y. Lin, J. Fu, K. Liu, C. Cai, Q. Wang, H. Li, X. Li, *Angew. Chem. Int. Ed.* **2021**, 60, 16607.

[30] L. Han, Y. Sun, S. Li, C. Cheng, C. E. Halbig, P. Feicht, J. L. Hübner, P. Strasser, S. Eigler, *ACS Catal.* **2019**, 9, 1283.

[31] G. Chen, J. Liu, Q. Li, P. Guan, X. Yu, L. Xing, J. Zhang, R. Che, *Nano Research* **2019**, 12, 2614.

[32] X. Xiao, T. Wang, J. Bai, F. Li, T. Ma, Y. Chen, *ACS Appl. Mater. Interfaces* **2018**, 10, 42534.

[33] W. Chen, H. Jin, F. He, P. Cui, C. Cao, W. Song, *Nano Research* **2022**, 15, 3017.

[34] J. Yu, C. Cao, H. Jin, W. Chen, Q. Shen, P. Li, L. Zheng, F. He, W. Song, Y. Li, *Natl. Sci. Rev.* **2022**, https://doi.org/10.1093/nsr/nwac018.

[35] J. Liu, C. Cao, X. Liu, L. Zheng, X. Yu, Q. Zhang, L. Gu, R. Qi, W. Song, *Angew. Chem. Int. Ed.* **2021**, 60, 15248.

[36] Q. Shen, P. Li, W. Chen, H. Jin, J. Yu, L. Zhu, Z. Yang, R. Zhao, L. Zheng, W. Song, *Sci. China Mater.* **2022**, 65, 163.

[37] H. Zhou, Y. Zhao, J. Gan, J. Xu, Y. Wang, H. Lv, S. Fang, Z. Wang, Z. Deng, X. Wang, P. Liu, W. Guo, B. Mao, H. Wang, T. Yao, X. Hong, S. Wei, X. Duan, J. Luo, Y. Wu, *J. Am. Chem. Soc.* **2020**, 142, 12643.

[38] Y. Pan, Y. Chen, K. Wu, Z. Chen, S. Liu, X. Cao, W.-C. Cheong, T. Meng, J. Luo, L. Zheng, *Nat. Commun.* **2019**, 10, 4290.

[39] Y. Zhu, W. Sun, J. Luo, W. Chen, T. Cao, L. Zheng, J. Dong, J. Zhang, M. Zhang, Y. Han, C. Chen, Q. Peng, D. Wang, Y. Li, *Nat. Commun.* **2018**, 9, 3861.

[40] T. Zhang, D. Zhang, X. Han, T. Dong, X. Guo, C. Song, R. Si, W. Liu, Y. Liu, Z. Zhao, *J. Am. Chem. Soc.* **2018**, 140, 16936.

[41] M. Zhang, Y.-G. Wang, W. Chen, J. Dong, L. Zheng, J. Luo, J. Wan, S. Tian, W.-C. Cheong, D. Wang, *J. Am. Chem. Soc.* **2017**, 139, 10976.

[42] D. Deng, X. Chen, L. Yu, X. Wu, Q. Liu, Y. Liu, H. Yang, H. Tian, Y. Hu, P. Du, R. Si, J. Wang, X. Cui, H. Li, J. Xiao, T. Xu, J. Deng, F. Yang, N. Duchesne Paul, P. Zhang, J. Zhou, L. Sun, J. Li, X. Pan, X. Bao, *Sci. Adv.* **2015**, 1, e1500462.

[43] J. Li, Y. Xu, Z. Ding, A. H. Mahadi, Y. Zhao, Y.-F. Song, *Chem. Eng. J* **2020**, 388, 124248.

[44] B. Lee, H. Naito, T. Hibino, *Angew. Chem. Int. Ed.* **2012**, 51, 440.

[45] H. Long, T.-S. Chen, J. Song, S. Zhu, H.-C. Xu, *Nat. Commun.* **2022**, 13, 3945.

[46] K. Jiang, S. Back, A. J. Akey, C. Xia, Y. Hu, W. Liang, D. Schaak, E. Stavitski, J. K. Nørskov, S. Siahrostami, H. Wang, *Nat. Commun.* **2019**, 10, 3997.
